# Non-symmetric responses of leaf onset date to natural warming and cooling in northern ecosystems

**DOI:** 10.1093/pnasnexus/pgad308

**Published:** 2023-09-19

**Authors:** Lei He, Jian Wang, Philippe Ciais, Ashley Ballantyne, Kailiang Yu, Wenxin Zhang, Jingfeng Xiao, François Ritter, Zhihua Liu, Xufeng Wang, Xiaojun Li, Shouzhang Peng, Changhui Ma, Chenghu Zhou, Zhao-Liang Li, Yaowen Xie, Jian-Sheng Ye

**Affiliations:** College of Earth and Environmental Sciences, Lanzhou University, Lanzhou 730000, China; State Key Laboratory of Efficient Utilization of Arid and Semi-arid Arable Land in Northern China, Institute of Agricultural Resources and Regional Planning, Chinese Academy of Agricultural Sciences, Beijing 100081, China; Department of Geography, The Ohio State University, Columbus, OH 43210, USA; Laboratoire des Sciences du Climat et de l′‌Environnement, CEA/CNRS/UVSQ/Université Paris Saclay, Gif-sur-Yvette 91191, France; Laboratoire des Sciences du Climat et de l′‌Environnement, CEA/CNRS/UVSQ/Université Paris Saclay, Gif-sur-Yvette 91191, France; Department of Ecosystem and Conservation Sciences, University of Montana, Missoula, MT 59801, USA; Laboratoire des Sciences du Climat et de l′‌Environnement, CEA/CNRS/UVSQ/Université Paris Saclay, Gif-sur-Yvette 91191, France; Department of Ecology & Evolutionary Biology, Princeton University, Princeton, NJ 08544, USA; Department of Physical Geography and Ecosystem Science, Lund University, Lund 22362, Sweden; Earth Systems Research Center, Institute for the Study of Earth, Oceans, and Space, University of New Hampshire, Durham, NH 03824, USA; Laboratoire des Sciences du Climat et de l′‌Environnement, CEA/CNRS/UVSQ/Université Paris Saclay, Gif-sur-Yvette 91191, France; CAS Key Laboratory of Forest Ecology and Management, Institute of Applied Ecology, Chinese Academy of Sciences, Shenyang 110016, China; Key Laboratory of Remote Sensing of Gansu Province, Heihe Remote Sensing Experimental Research Station, Northwest Institute of Eco-Environment and Resources, Chinese Academy of Sciences, Lanzhou 730000, China; INRAE, UMR1391 ISPA, Université de Bordeaux, Villenave d′‌Ornon 33140, France; State Key Laboratory of Soil Erosion and Dryland Farming on the Loess Plateau, Northwest A&F University, Yangling 712100, China; State Key Laboratory of Efficient Utilization of Arid and Semi-arid Arable Land in Northern China, Institute of Agricultural Resources and Regional Planning, Chinese Academy of Agricultural Sciences, Beijing 100081, China; Center for Ocean Remote Sensing of Southern Marine Science and Engineering Guangdong Laboratory (Guangzhou), Guangzhou Institute of Geography, Guangdong Academy of Sciences, Guangzhou 510070, China; State Key Laboratory of Efficient Utilization of Arid and Semi-arid Arable Land in Northern China, Institute of Agricultural Resources and Regional Planning, Chinese Academy of Agricultural Sciences, Beijing 100081, China; State Key Laboratory of Resources and Environmental Information System, Institute of Geographic Sciences and Natural Resources Research, Chinese Academy of Sciences, Beijing 100101, China; College of Earth and Environmental Sciences, Lanzhou University, Lanzhou 730000, China; Key Laboratory of Western China's Environmental Systems (Ministry of Education), Lanzhou University, Lanzhou 730000, China; State Key Laboratory of Herbage Improvement and Grassland Agro-Ecosystems, College of Ecology, Lanzhou University, Lanzhou 730000, China

## Abstract

The northern hemisphere has experienced regional cooling, especially during the global warming hiatus (1998–2012) due to ocean energy redistribution. However, the lack of studies about the natural cooling effects hampers our understanding of vegetation responses to climate change. Using 15,125 ground phenological time series at 3,620 sites since the 1950s and 31-year satellite greenness observations (1982–2012) covering the warming hiatus period, we show a stronger response of leaf onset date (LOD) to natural cooling than to warming, i.e. the delay of LOD caused by 1°C cooling is larger than the advance of LOD with 1°C warming. This might be because cooling leads to larger chilling accumulation and heating requirements for leaf onset, but this non-symmetric LOD response is partially offset by warming-related drying. Moreover, spring greening magnitude, in terms of satellite-based greenness and productivity, is more sensitive to LOD changes in the warming area than in the cooling. These results highlight the importance of considering non-symmetric responses of spring greening to warming and cooling when predicting vegetation-climate feedbacks.

**Significance Statement**

Regional temperature decrease (i.e. cooling) was observed during the global warming hiatus (1998–2012), yet its influence on the spring greening with earlier leaf onset date and higher productivity remains unknown. Using ground observations, remote sensing imagery, and model estimates, here, we show that the response of spring greening to temperature is nonlinear, with a stronger response to natural cooling than to warming. The future projection indicates a stronger impact of warming than cooling, leading to a larger uncertainty of vegetation–climate feedbacks. This study challenges the notion of linear temperature sensitivity and contributes to future model projections.

## Introduction

Spring leaf onset date (LOD) has advanced in recent decades in northern mid to high latitudes (>30°N) under global warming (1–7). This advance is highly sensitive to temperature changes, extends the growing season length, and accordingly increases the carbon uptake of terrestrial ecosystems (1, 8, 9). Understanding the responses of LOD to temperature changes in terms of sign and magnitude is therefore crucial for assessing the influence of climate change on terrestrial ecosystems and its feedback to climate (10, 11). Unlike warming effects, however, most existing studies ignore the impact of cooling anomalies on LOD, which may cause biased predictions of vegetation–climate feedbacks (12).

In northern regions, winter and spring temperatures are generally considered the principal drivers of spring LOD. Trees need to accumulate enough winter chilling to end the endodormancy phase and enough spring warming to break the ecodormancy phase, further triggering plant leaf onset (13–16). The earlier emergence of spring leaves has been associated with warmer temperatures because of easily reaching heating demand (14, 17). However, the global mean temperature has not always shown a steady increase, with the global warming hiatus observed between 1998 and 2012 possibly due to an energy redistribution within the oceans (18–21). Until now, we only know the relative responses of LOD to warming and cooling for some species, e.g. tree saplings and grass, in field experiments (12, 22, 23). Warming of 1°C in winter/spring led to an advance of 8.8 days in budburst dates of *Fagus sylvatica L.*, whereas 1°C cooling delayed it by 10.9 days (22). Two manipulative experiments in the Tibetan Plateau showed non-significant differences in sensitivities to warming and cooling for grass leaf-out (12, 23). Both field- and ecosystem-scale analyses have mainly focused on advancing effects of natural warming on LOD, influenced by photoperiod (24, 25), precipitation amount (26, 27) and frequency (28), and soil water (29) and nutrient availability (30). Besides direct physiological effects, temperature changes regulate ecosystem composition and function, soil moisture, snowmelt, and permafrost degradation in high altitudes and latitudes, which may affect plant spring growth (31–35). There is limited evidence of non-symmetric or symmetric responses of LOD to natural warming and cooling at the species to ecosystem scales, especially in mature woody biomes (23). The impacts of spring greening timing (i.e. LOD) on spring greening magnitudes (i.e. spring greenness and productivity) during the warming hiatus are also unclear. Therefore, we ask two questions: (i) does spring LOD respond to warming and cooling symmetrically in northern biomes? and (ii) what are the physical and physiological mechanisms related to non-symmetric or symmetric patterns? To this end, we investigated the responses of LOD to natural warming and cooling, using gridded meteorological data (temperature, precipitation, and cloud cover) together with LOD data from two independent data sets: (i) 15,125 ground phenological time series at 3,620 sites across Europe since the 1950s and (ii) normalized difference vegetation index (NDVI) data, for northern mid to high latitudes (>30°N) from 1982 to 2012.

## Responses of LOD to natural warming and cooling

**Fig. 1.**
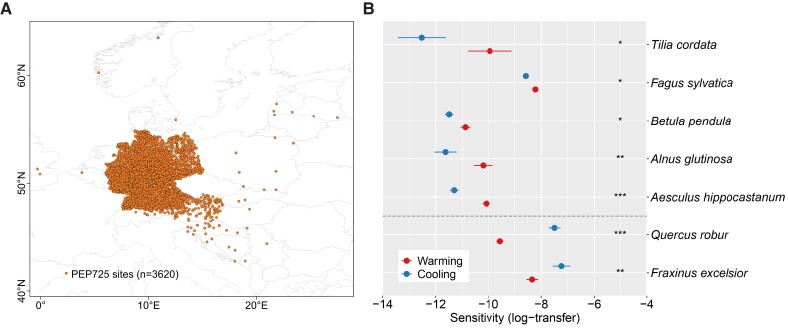
The distribution of PEP725 sites and comparisons of LOD responses to warming and cooling at the species scale. A) The locations of PEP725 sites for long-term in situ LOD observations of seven European dominant tree species since the 1950s. B) LOD responses (log-transfer, see Methods) to warming and cooling at the species scale from PEP725 ground observation data. The warming and cooling samples were obtained by using *P* < 0.05 for temperature changes and partial correlation analysis. The bar represents the standard error. Student's *t*-test was used to test the significance of the difference between the warming and cooling conditions. Significance code for differences: ***P < 0.001, **P < 0.01, and *P < 0.05.

We used long-term in situ LOD observations of seven European dominant tree species (5), derived from the PEP725 database that provides the longest and most comprehensive phenological records, to study the LOD responses to warming and cooling (Fig. 1) (see Methods). The results overall indicated the non-symmetric LOD responses to warming and cooling, i.e. five out of seven species (*Tilla cordata P* < 0.05, *Fagus sylvatica P* < 0.05, *Betula pendula P* < 0.05, *Alnus glutinosa P* < 0.01, and *Aesculus hippocastanum P* < 0.001) were more sensitive to cooling than to warming (Fig. 1B). Consistent with previous field experiment for *Fagus sylvatica* (22), our results highlight the stronger responses of LOD to natural cooling than to warming, indicating a nonlinear temperature control of LOD.

**Fig. 2.**
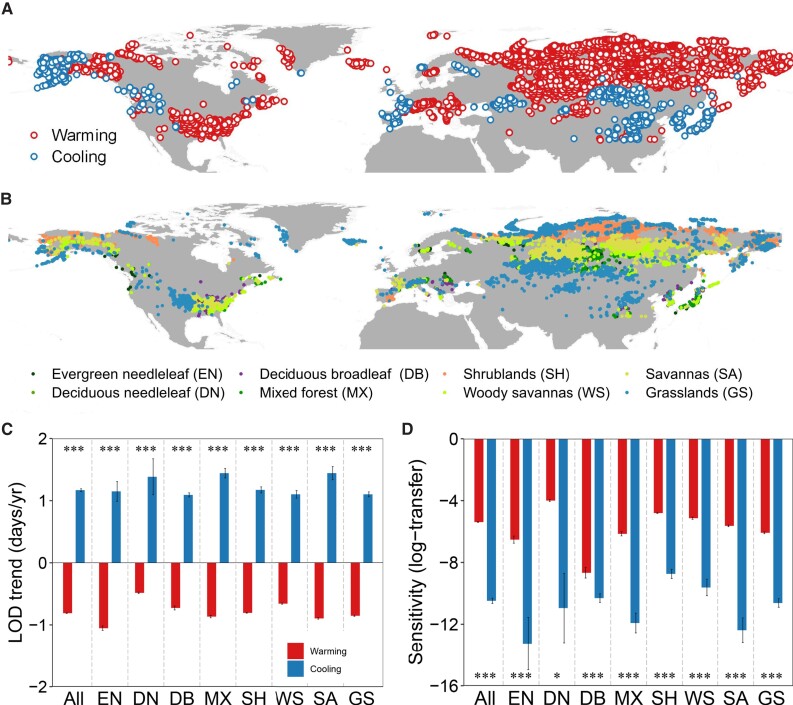
Warming and cooling grid cells used in this study and comparisons of LOD responses to warming and cooling at the biome scale. A) Locations of warming and cooling grid cells during the warming hiatus (1998–2012). Grid cells with *P* < 0.05 for temperature changes and partial correlation analysis were retained (see Methods). B) The biome types of grid cells. C) Trends in LOD in warming and cooling areas for biomes from 1998 to 2012. D) LOD responses (log-transfer) to warming and cooling at the biome scale from satellite-based LOD data. The bar represents the standard error. Student's *t*-test was used to test the significance of the difference between the warming and cooling conditions. Significance code for differences: ***P < 0.001, **P < 0.01, and *P < 0.05.

To focus on the spatial comparison of LOD responses within the biomes, we applied the warming hiatus period (1998–2012) to identify warming and cooling grid cells for satellite-based analysis by using statistical significance at the 0.05 level for temperature changes and partial correlation analysis (see Methods) (Fig. 2A, B). Results showed that LOD advanced in the warming areas and delayed in the cooling areas for all the forest and grass biomes between 1998 and 2012 (Fig. 2C). Overall, the magnitudes of LOD response to cooling were greater than those to warming, that is, the sensitivity (log transformation) of LOD to warming and cooling was −5.4 ± 0.02 (mean ± SE, standard error) vs. −10.5 ± 0.2 (*P* < 0.001). All forest and grass biomes showed consistently non-symmetric LOD responses to warming and cooling, i.e. the sensitivity (log transformation) of LOD to warming and cooling was −6.5 ± 0.2 vs. −13.3 ± 1.7 (*P* < 0.001), −4.00 ± 0.06 vs. −11.0 ± 2.3 (*P* < 0.05), −8.7 ± 0.4 vs. −10.3 ± 0.3 (*P* < 0.001), −6.1 ± 0.1 vs. −11.9 ± 0.6 (*P* < 0.001), −4.8 ± 0.02 vs. −8.7 ± 0.3 (*P* < 0.001), −5.1 ± 0.07 vs. −9.6 ± 0.5 (*P* < 0.001), −5.6 ± 0.05 vs. −12.4 ± 0.8 (*P* < 0.001), and −6.1 ± 0.06 vs. −10.6 ± 0.3 (*P* < 0.001) for evergreen needleleaf forests, deciduous needleleaf forests, deciduous broadleaf forests, mixed forests, shrublands, woody savannas, savannas, and grasslands, respectively (Fig. 2D). The results using the significance level of 0.01 for temperature changes and partial correlation analysis were consistent with the 0.05 level (Fig. S1; Tables S1 and S2). In addition to spatial analysis, we also performed a temporal analysis by comparing the LOD responses to temperature changes for grids that experienced warming during 1982–97 and cooling during 1998–2012 (Fig. S2). The temporal result supported that the magnitudes of LOD response to cooling were greater than those to warming.

To quantify the variations of non-symmetric LOD response, we defined a LOD non-symmetric index calculated by the difference between the LOD sensitivities to warming and cooling; the positive value indicates stronger sensitivity to cooling, while the negative value suggests stronger sensitivity to warming (see Methods). Using the LOD simulated by a growing-degree-day (GDD) algorithm (see Methods), we also found that LOD was more sensitive to cooling than to warming during the warming hiatus (Fig. S3). For future projections (2016–99), we also found a non-systematic response but with different patterns, i.e. LOD becomes more sensitive to warming than to cooling under the scenarios of highest baseline of carbon emissions (RCP8.5) (Fig. S4).

## The mechanisms under non-symmetric LOD responses

**Fig. 3.**
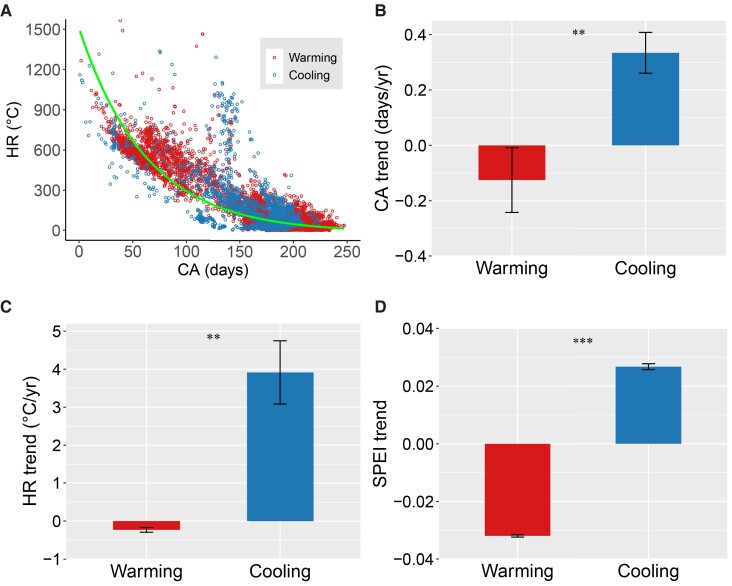
Comparisons of changes for chilling accumulation (CA), heat requirement (HR), and water availability indicator (i.e. SPEI) in warming and cooling areas obtained from satellite-based analysis during the warming hiatus. A) Relationship between CA and HR. The green line indicates an exponential decay regression fitted using CA and HR. B, C, and D) present the trends of CA, HR, and SPEI in the warming and cooling areas during 1998–2012, respectively. The bar represents the standard error. Student's *t*-test was used to test the significance of the difference in the absolute values of trends for CA, HR, and SPEI in the warming and cooling areas. Significance code for differences: ***P < 0.001 and **P < 0.01.

Exploring physiological mechanisms under LOD responses is challenging. Here, we hypothesized that the non-symmetric LOD responses to warming and cooling might be related to changes in chilling accumulation (CA, the amount of chilling during endodormancy), heat requirement (HR, the accumulated forcing temperature required for leaf onset), and water availability (16, 36–38). To test these hypotheses, we determined the changes in CA, HR, and water stress using a drought index (the Standardized Precipitation Evapotranspiration Index, SPEI) in warming and cooling areas during the warming hiatus (see Methods). First, we confirmed a dual role of temperature in controlling LOD variations with an exponential decay-like relationship between chilling days and forcing degrees (16) (Fig. 3A). Grouping grid cells into warming and cooling indicated that warming reduced CA and HR while cooling increased the two variables (Fig. 3B, C; Figs. S5–S6). Trees with more CA in the phase of endodormancy might need more HR to break ecodormancy for reactivating growth (13, 39). Expectedly, cooling grids showed more changes in HR caused than warming grids both in terms of trees (forests) and low vegetation (shrublands, savannas, woody savannas, and grasslands) (Fig. 3C; Figs. S6–S7). Non-symmetric changes in CA and HR caused by warming and cooling might follow the non-symmetric LOD responses.

On the other hand, we found that warming and cooling are associated with soil water availability changes (i.e. SPEI trend), further affecting LOD when controlling the effects of precipitation and radiation (Fig. S8). Decreased SPEI generally accompanies abundant sunshine in the warming areas, and these processes together lead to earlier LOD (40–43) (Fig. S8). In the current climate, the severity of preseason drying may not reach a turning point that could cause a delaying effect on LOD (40). Before the turning point, the elevated preseason temperature and radiation in drought may advance LOD (3, 40, 44). In contrast, cooling benefited maintaining soil water availability (Fig. S8A), offsetting the advancing effect caused by drought stress (40, 41) and leading to delayed LOD (Fig. S8B). We found stronger effects of warming on water availability than effects of cooling (Fig. 3D). Considering the opposite and non-symmetric effects on soil water availability, the non-symmetric LOD responses to warming and cooling might be partially offset.

For future projections (2016–99), we found a reversion of warming and cooling effect sizes, that is LOD will be more sensitive to warming than to cooling under RCP8.5 (Fig. S4B). Apart from the projection uncertainty caused by models and datasets, we proposed two potential reasons. First, future climate change may alter current non-symmetric patterns of chilling accumulation and heating requirements under warming and cooling, especially with temperature increases and precipitation variations (3, 28). Second, warming-related drying stress might adjust climatic responses leading to a warming-dominant control on spring plant growth (45–47).

## Connections among temperature change, LOD, and spring greening magnitude

**Fig. 4.**
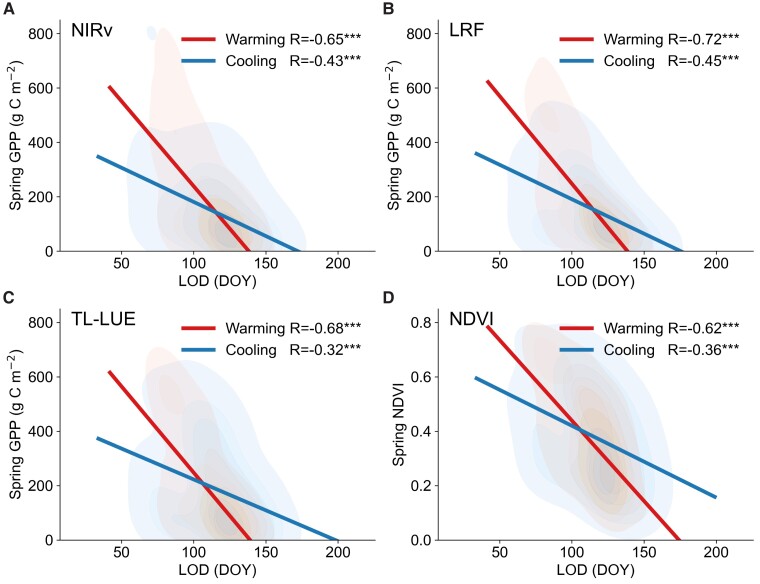
Connections among temperature change, LOD, and spring greening magnitude. The comparison for regressions between spring GPP/NDVI and LOD in the warming and cooling areas using the satellite-based NIRv GPP A), the LRF GPP B), TL-LUE GPP C), and GIMMS NDVI D), respectively. The spring-accumulated GPP and mean NDVI were calculated during the period from March to May. The red and blue kernel density plots represent the density distribution of warming and cooling grids in GPP/NDVI-LOD space, respectively. The four GPP/NDVI datasets all showed that the differences in the slopes between warming and cooling conditions were significant (*P* < 0.001) by using covariance analysis. Significance code for differences: ***P < 0.001.

Spring (from March to May) accumulated gross primary productivity (GPP) and mean NDVI were used as proxies of spring greening magnitude during the warming hiatus. Spring greening magnitudes were negatively correlated with spring greening timing (i.e. LOD) (Fig. 4), which suggested that earlier LOD would increase plant carbon uptake in spring (40, 48, 49). However, these relationships were significantly different in the warming and cooling areas by using covariance analysis (*P* < 0.001) (50). We also used the random slope model to present the relationship between spring GPP/NDVI and LOD in the temporal scale when controlling for latitudes and longitudes of grid cells as random factors (Fig. S9). To reduce the uncertainty brought by the fixed period used (i.e. from March to May), we calculated the spring greening magnitude with accumulated GPP and mean NDVI during the period from LOD to the maturity (i.e. the date corresponding to the maximum NDVI in the GIMMS NDVI3g time series) and obtained the similar results with fixed period used (Fig. S10). As confirmed by these independent lines of evidence, spring GPP/NDVI was more sensitive to LOD in the warming areas than in the cooling (Fig. 4; Figs. S9–S10), suggesting that the increase in spring plant productivity caused by 1-day LOD advance by warming was overall greater than the decrease by 1-day LOD delay by cooling. These results call for caution concerning model-based climatic responses of GPP and aid in understanding vegetation–climate feedbacks.

## Conclusions

Using both ground records and satellite observations, we found non-symmetric LOD responses to natural warming and cooling, i.e. the LOD of northern biomes exhibited stronger responses to cooling than to warming. The underlying mechanism might be associated with stronger variations of CA and HR by cooling, which could be partially offset by warming-associated drying. Moreover, the spring greening magnitude was more sensitive to LOD changes in the warming areas than in the cooling. Our findings provide a new conceptual framework of LOD responses to climate change, which is enlightening for model improvements and projections.

## Methods

### In situ LOD observation

We applied the in situ LOD observations from the Pan European Phenology Project (PEP725), which is an open database that contains long-term plant phenological observations from 25 European countries (http://www.pep725.eu/) (51). The date of the first visible foliar stalk for tree species (BBCH code 11) was used. All available records (15,125) from 1951 to 2018 were collected from 3,620 sites for seven European dominant tree species (5), i.e. *Aesculus hippocastanum*, *Alnus glutinosa*, *Betula pendula*, *Fagus sylvatica*, *Fraxinus excelsior*, *Quercus robur*, and *Tilia cordata* (Fig. 1A).

### Satellite greenness–based LOD

We used Global Inventory Modeling and Mapping Studies (GIMMS) NDVI3g v1 data to derive LOD between 1982 and 2012 (52). The NDVI3g v1 data are derived from optical surface reflectance measurements taken by a series of NOAA-AVHRR satellites. Corrections for intersensor calibration, orbital drifts, and stratospheric aerosols from volcanic eruptions have made it the most consistent long-term satellite vegetation dataset currently available (52, 53). To remove snow effects, we replaced all contaminated NDVI with the mean of snow-free NDVI values from all years in winter (December–February) (54). A modified Savitzky–Golay filter was then used to eliminate abnormal values and reconstruct the NDVI time series (55). Furthermore, we eliminated sparse vegetation by removing grids with a mean annual NDVI value of less than 0.1. We applied two methods, i.e. the dynamic threshold approach and the double-logistic function, to estimate LOD to minimize the uncertainty from a single method. The two methods show similar results, so we calculated the average LOD from the two methods as the final LOD.

In the first method, we calculated NDVI ratios annually for each pixel as follows:


(1) $${\mathrm{NDVI}}_{\mathrm{ratio}}=\frac{{\mathrm{NDVI}}_{\mathrm{day}}\;\text{–}\;{\mathrm{NDVI}}_{\min}}{{\mathrm{NDVI}}_{\max}\;\text{–}{\;\mathrm{NDVI}}_{\min}},$$

where ${\mathrm{NDVI}}_{\mathrm{day}}$ is daily NDVI and ${\mathrm{NDVI}}_{\min}$ and ${\mathrm{NDVI}}_{\max}$ are the minimum and maximum NDVI of each year, respectively. A threshold ratio of 0.5 was used to determine LOD.

In the second method, we fitted the NDVI time series with a double-logistic function and then calculated the second-order derivative of the fitted curve. LOD was defined as the time when the rate of change in curvature reached its first local maximum in spring.


(2) $$y(t)\;=\;a\;+\;b\left(\frac{1}{1+e^{c(t-d)}}\;+\;\frac{1}{1+e^{e(t\;-f)}}\right),$$

where *t* is time in days and *y*(*t*) is the NDVI value at time *t*. *a* is the initial background NDVI value, and *b* − *e* are parameters of this function.

## Spring greening magnitude

We used mean NDVI and accumulated GPP from March to May as spring greening magnitude. The GIMMS NDVI3g v1 dataset was used to calculate spring mean NDVI. We used GPP data from three independent sources, i.e. the monthly satellite-based near-infrared reflectance (NIRv) GPP, the daily light response function (LRF) GPP, and the 8-day two-leaf light use efficiency model (TL-LUE) GPP datasets. The NIRv GPP dataset has good performance at capturing seasonal and interannual variations of terrestrial GPP at a global scale (56). LRF GPP was estimated by an ecosystem-level physiological method using an asymptotic light response function between incoming photosynthetically active radiation (PAR) and GPP, which well represents the response observed at high spatiotemporal resolutions (57). TL-LUE GPP dataset distinguished GPP of sunlit and shaded leaves, suitable for studying the variations in seasonal cycles of GPP over many years (58).

## Climatic data

In satellite-derived analysis, we used Multi-Source Weather (MSWX) temperature, precipitation, and radiation data with daily temporal resolution and 0.1° spatial resolution (59) in partial correlation analysis to determine optimal preseason length and the site-specific period before LOD with the highest absolute partial-correlation coefficient (see Analyses). European gridded observational (E-OBS v23.1e) daily climate data with a spatial resolution of 0.1° were used in the phenological observation analysis at the species scale with PEP725 data. This dataset was provided by the ECA&D (European Climate Assessment & Dataset) project (60).

The daily temperature of the GFDL-ESM2M model in ISIMIP2b (Inter-Sectoral Impact Model Intercomparison Project 2b simulation round) was used to predict LOD from 2016 to 2099 under future scenarios (RCP4.5 and RCP8.5), and surface downwelling shortwave radiation and precipitation data were used to calculate the LOD responses to warming and cooling.

## Chilling and forcing models

We used 10 chilling models to measure the number of chilling days (i.e. CA) and 8 GDD models for estimating the heating requirement (HR) for the LOD. Chilling models 1–8, 11, and 12 and GDD models 1–8 in (16) were used in our study.

## Model for predicting LOD

To predict future LOD, we used a two-phase parallel model (PM) with optimal parameters (61). In contrast to the one-phase models (e.g. growing degree days [GDD] and spring warming [SW] models) that focus only on forcing accumulation, PM assumes that the forcing accumulation cannot begin until a critical threshold (*C*_crit_) of the chilling state (*S*_c_, daily sum of chilling rates) is reached (62). The first phase of PM is chilling accumulation. A triangle function (Eq. 3) was used to describe the daily rate of chilling (*R*_c_) (63), and *S*_c_ began to accumulate after September 1^st^ of the preceding year (*t*_c_) (Eq. 4):


(3) $$R_{c}\;=\;\{\;\begin{array}{cc} 0, & T\;\leq \;T_{a}\\ \frac{T-T_{a}}{T_{b}-T_{a}}, & T_{a}\;<\;T\;\leq \;T_{b}\\ \frac{T-T_{c}}{T_{b}-T_{c}}, & T_{b}\;<\;T\;<\;T_{c}\\ 0, & T\;\geq \;T_{c} \end{array}$$

(4) $$S_{c}\;=\;\overset{t}{\underset{t_{c}}{\sum }}\;R_{c},$$

where $R_{c}$ is the daily rate of chilling. *T* is the daily mean temperature. *T*_a_, *T*_b_, and *T*_c_ are three model parameters. *S*_c_ is the daily sum of chilling rates and begins to accumulate after September 1^st^ of the preceding year ($t_{c}$).

The second phase of PM is forcing accumulation, and the day that the state of force ($S_{f}$) achieved its critical value (*F*_crit_) was used to determine the modeled LOD.


(5) $$S_{f}\;=\overset{t}{\underset{t_{0}}{\sum }}\;R_{f}$$

(6) $$R_{f}=\{\begin{array}{cc} 0, & T\;\leq T_{d}\\ K\;\times \;\frac{A_{f}}{1+e^{\mathrm{alpha}\;\times \;(T\text{-}\mathrm{beta})},} & T\;>\;T_{d} \end{array}$$

(7) $$K\;=\;\{\begin{array}{cc} K_{\min}\;+\;\frac{1\;-K_{\min}}{C_{\mathrm{crit}}}\;\times \;S_{c}, & S_{c}\;<\;C_{\mathrm{crit}}\\ 1, & S_{c}\;\geq \;C_{\mathrm{crit}} \end{array}$$

(8) $$\mathrm{LOD}=t,\;\;\mathrm{if}\;\;S_{f}\;\geq \;F_{\mathrm{crit}},$$

where $R_{f}$ is the daily rate of forcing and starts from January 1^st^ of the current year (*t*_0_). $T_{d}$ is a temperature threshold to establish the requirement for beginning the accumulation of forcing (Eq. 6) and fulfilling *C*_crit_. *A*_f_, alpha, beta, *F*_crit_, and *C*_crit_ are model parameters. *K* is an adjustment factor to ensure that the accumulation of forcing occurs after the chilling state (*C*_crit_) is fulfilled. *K*_min_ is another model parameter that determines the minimum potential of an unchilled bud to respond to the forcing temperature (63). Finally, the date when *S*_f_ exceeds *F*_crit_ is regarded as the LOD (Eq. 8).

PM parameters, including *A*_f_, alpha, beta, *F*_crit_, *C*_crit_, *T*_a_, *T*_b_, *T*_c_, *T*_d_, and *K*_min_, were calibrated optimally by implementing the particle swarm optimization (PSO) algorithm (SPSO-2011) at each pixel, based on 31 years of satellite-derived LOD (1982–2012) and gridded air temperature data with daily scale. The set of optimal parameters was employed when the RMSE value between the observed and modeled LOD was the lowest. With the optimal parameters, we used PM to predict future LOD under scenarios RCP4.5 and RCP8.5. It should be noted that the uncertainty of future climatic projections might, to some degree, undermine the robustness of future LOD and its responses to warming and cooling.

## Analyses

We performed ground- and satellite-based analyses at the species and biome scales, respectively. We used seven dominant tree species with long-term in situ observations of LOD in Europe from the PEP725 database. The biome types were obtained based on MCD12C1 land cover product (64).

To identify warming and cooling periods/grid cells, PEP725 phenology and E-OBS climate data were applied for ground-based analysis. The satellite-derived LOD and MSWX climate data were employed for the biome-based analysis. The relevant periods for preseason temperature impacts on phenology vary among biomes, species, and locations (37). To determine the optimal preseason during which average temperature had the largest influence on phenology, we computed the partial correlation coefficients between average temperature and LOD, controlling the effects of precipitation and radiation, from 0 to 6 months before the mean LOD with a step of 8 days (65). The optimal preseason length was the period with the highest absolute partial correlation coefficient. We then calculated the temperature trend during the optimal preseason length by linear least-squares regression analysis with year as the independent variable. This study used statistical significance at a 0.05 level for partial correlation and trend analysis. Due to the different time lengths of PEP725 records, we used a 15-year moving window to obtain warming and cooling periods in the time series of each station. In the satellite-based analysis, we applied the warming hiatus period (1998–2012) to identify warming and cooling grid cells. In addition, we identified grid cells with warming during 1982–98 and cooling during 1998–2012 for temporal analysis. For future projections, we determined the temperature-relevant preseason and computed the temperature changes during preseason for all grids within a moving window of 15 years and then identified the warming and cooling grids in each moving window from 2016 to 2099 based on ISIMIP2b climatic datasets. Finally, we calculated the non-symmetric index within a moving window of 15 years (Fig. S4).

Due to nonlinear temperature responses, the LOD responses to temperature changes were computed by log–log regression to avoid potential statistical artifacts using the linear method (66, 67). In the ground-based analysis, we calculated the average value of the LOD responses to warming and cooling in each station and then the average value of the LOD responses to warming and cooling in each species. In the satellite-based analysis, we calculated the average value of the LOD responses to warming and cooling for each biome at the spatial (warming vs. cooling during 1998–2012) and temporal (warming during 1982–98 vs. cooling during 1998–2012) scales. Finally, the non-symmetric LOD response to warming and cooling was determined by Student's *t*-test method (at least *P* < 0.05).

We compared the changes in the number of chilling days, accumulated forcing degrees, and a water indicator at the warming and cooling grids during 1998–2012 to explore the mechanisms under the non-symmetric/symmetric LOD responses to warming and cooling. To calculate the water indicator, we employed monthly SPEI data at a spatial resolution of 0.5° from the SPEI base v. 2.7 at Consejo Superior de Investigaciones Científicas (CSIC) (68). The SPEI data consisted of multiscale monthly SPEI from 1 to 48 months; we selected the 3-month SPEI to capture the short-term water deficit (69). We calculated trends of chilling days, forcing degrees, and SPEI in the relevant periods for preseason temperature by the linear least-squares regression method. Then, we used Student's *t*-test method to check whether non-symmetric patterns exist.

We applied GPP and NDVI data to compute spring greening magnitude between March and May from 1998 to 2012. The regressions between spring greening magnitude and LOD in the warming and cooling conditions were created by the least-squares linear regression method. Then, we tested the statistical significance of the difference in the slopes of GPP/NDVI-LOD regressions between warming and cooling conditions by covariance analysis based on a procedure in (50). Finally, we applied a random slope model (“lme4” package in R4.2.0) to compare GPP/NDVI-LOD regressions in warming and cooling scenarios at the temporal scale when grid cells’ latitudes and longitudes were used as random factors.

## Supplementary Material

pgad308_Supplementary_Data

## Data Availability

All study data are public and included in supplementary information. The specific links for data used in this study can be found in Table S3.

## Code availability

The codes used for data analysis are available on Zenodo at https://doi.org/10.5281/zenodo.7859244.

## References

[1] Keenan TF , et al 2014. Net carbon uptake has increased through warming-induced changes in temperate forest phenology. Nat Clim Change. 4:598–604.

[2] Menzel A , et al 2006. European Phenological response to climate change matches the warming pattern. Glob Change Biol. 12:1969–1976.

[3] Piao S , et al 2015. Leaf onset in the northern hemisphere triggered by daytime temperature. Nat Commun. 6:6911. 25903224 10.1038/ncomms7911 PMC4423217

[4] Park T , et al 2016. Changes in growing season duration and productivity of northern vegetation inferred from long-term remote sensing data. Environ Res Lett. 11:084001.

[5] Fu YH , et al 2015. Declining global warming effects on the phenology of spring leaf unfolding. Nature. 526:104–107. 26416746 10.1038/nature15402

[6] Gu H , et al 2022. Warming-induced increase in carbon uptake is linked to earlier spring phenology in temperate and boreal forests. Nat Commun. 13:3698. 35760820 10.1038/s41467-022-31496-w PMC9237039

[7] Shen M , et al 2022. Plant phenology changes and drivers on the Qinghai–Tibetan Plateau. Nat Rev Earth Environ. 3:633–651.

[8] Myneni RB , Keeling CD, Tucker CJ, Asrar G, Nemani RR. 1997. Increased plant growth in the northern high latitudes from 1981 to 1991. Nature. 386:698–702.

[9] Zhou Y . 2022. Understanding urban plant phenology for sustainable cities and planet. Nat Clim Change. 12:302–304.

[10] Peñuelas J , Rutishauser T, Filella I. 2009. Phenology feedbacks on climate change. Science. 324:887–888. 19443770 10.1126/science.1173004

[11] Richardson AD , et al 2013. Climate change, phenology, and phenological control of vegetation feedbacks to the climate system. Agric For Meteorol. 169:156–173.

[12] Li X , et al 2016. Responses of sequential and hierarchical phenological events to warming and cooling in alpine meadows. Nat Commun. 7:12489. 27535205 10.1038/ncomms12489 PMC4992149

[13] Perry TO . 1971. Dormancy of trees in winter. Science. 171:29–36. 17737985 10.1126/science.171.3966.29

[14] Piao S , et al 2019. Plant phenology and global climate change: current progresses and challenges. Glob Change Biol. 25:1922–1940. 10.1111/gcb.14619 30884039

[15] Richardson AD , et al 2018. Ecosystem warming extends vegetation activity but heightens vulnerability to cold temperatures. Nature. 560:368–371. 30089905 10.1038/s41586-018-0399-1

[16] Wang H , et al 2020. Overestimation of the effect of climatic warming on spring phenology due to misrepresentation of chilling. Nat Commun. 11:4945. 33009378 10.1038/s41467-020-18743-8 PMC7532433

[17] Keenan TF . 2015. Spring greening in a warming world. Nature. 526:48–49. 26416739 10.1038/nature15633

[18] Medhaug I , Stolpe MB, Fischer EM, Knutti R. 2017. Reconciling controversies about the ‘global warming hiatus’. Nature. 545:41–47. 28470193 10.1038/nature22315

[19] Wang X , et al 2019. No trends in spring and autumn phenology during the global warming hiatus. Nat Commun. 10:2389. 31160586 10.1038/s41467-019-10235-8 PMC6546754

[20] Ballantyne A , et al 2017. Accelerating net terrestrial carbon uptake during the warming hiatus due to reduced respiration. Nat Clim Change. 7:148–152.

[21] Yan XH , et al 2016. The global warming hiatus: slowdown or redistribution? Earth’s Future. 4:472–482. 31423452 10.1002/2016EF000417 PMC6686362

[22] Signarbieux C , et al 2017. Asymmetric effects of cooler and warmer winters on beech phenology last beyond spring. Glob Change Biol. 23:4569–4580. 10.1111/gcb.13740 28464396

[23] Meng F , et al 2019. Divergent responses of community reproductive and vegetative phenology to warming and cooling: asymmetry versus symmetry. Front Plant Sci. 10:1310. 31681391 10.3389/fpls.2019.01310 PMC6811613

[24] Fu YH , et al 2019. Short photoperiod reduces the temperature sensitivity of leaf-out in saplings of Fagus sylvatica but not in horse chestnut. Glob Chang Biol. 25:1696–1703. 30779408 10.1111/gcb.14599

[25] Meng L , et al 2021. Photoperiod decelerates the advance of spring phenology of six deciduous tree species under climate warming. Glob Chang Biol. 27:2914–2927. 33651464 10.1111/gcb.15575

[26] Fu YH , et al 2014. Unexpected role of winter precipitation in determining heat requirement for spring vegetation green-up at northern middle and high latitudes. Glob Change Biol. 20:3743–3755. 10.1111/gcb.12610 24753114

[27] Shen M , Piao S, Cong N, Zhang G, Jassens IA. 2015. Precipitation impacts on vegetation spring phenology on the Tibetan Plateau. Glob Change Biol. 21:3647–3656. 10.1111/gcb.12961 25926356

[28] Wang J , Liu D, Ciais P, Peñuelas J. 2022. Decreasing rainfall frequency contributes to earlier leaf onset in northern ecosystems. Nat Clim Change. 12:386–392.

[29] Jolly WM , Running SW. 2004. Effects of precipitation and soil water potential on drought deciduous phenology in the Kalahari. Glob Change Biol. 10:303–308.

[30] Wang X , et al 2020. Satellite-observed decrease in the sensitivity of spring phenology to climate change under high nitrogen deposition. Environ Res Lett. 15:094055.

[31] Livensperger C , et al 2016. Earlier snowmelt and warming lead to earlier but not necessarily more plant growth. AoB Plants. 8:plw021. 27075181 10.1093/aobpla/plw021 PMC4866651

[32] Natali SM , Schuur EAG, Rubin RL. 2012. Increased plant productivity in Alaskan tundra as a result of experimental warming of soil and permafrost. J Ecol. 100:488–498.

[33] Cleland EE , Chiariello NR, Loarie SR, Mooney HA, Field CB. 2006. Diverse responses of phenology to global changes in a grassland ecosystem. Proc Natl Acad Sci U S A. 103:13740–13744. 16954189 10.1073/pnas.0600815103 PMC1560087

[34] Cleland EE , Chuine I, Menzel A, Mooney HA, Schwartz MD. 2007. Shifting plant phenology in response to global change. Trends Ecol Evol. 22:357–365. 17478009 10.1016/j.tree.2007.04.003

[35] Wang J , Liu D. 2022. Vegetation green-up date is more sensitive to permafrost degradation than climate change in spring across the northern permafrost region. Glob Change Biol. 28:1569–1582. 10.1111/gcb.16011 34854170

[36] Meng L , et al 2020. Urban warming advances spring phenology but reduces the response of phenology to temperature in the conterminous United States. Proc Natl Acad Sci U S A. 117:4228–4233. 32041872 10.1073/pnas.1911117117 PMC7049132

[37] Wu C , et al 2021. Widespread decline in winds delayed autumn foliar senescence over high latitudes. Proc Natl Acad Sci U S A. 118:e2015821118. 10.1073/pnas.2015821118 PMC8072329 33846246

[38] Wu C , et al 2018. Contrasting responses of autumn-leaf senescence to daytime and night-time warming. Nat Clim Change. 8:1092–1096.

[39] Lang GA . 1987. Dormancy: a new universal terminology. HortScience. 25:817–820.

[40] Zeng Z , et al 2021. Legacy effects of spring phenology on vegetation growth under preseason meteorological drought in the northern hemisphere. Agric For Meteorol. 310:108630.

[41] Deng H , Yin Y, Wu S, Xu X. 2019. Contrasting drought impacts on the start of phenological growing season in Northern China during 1982–2015. Int J Climatol. 40:3330–3347.

[42] Dai A . 2011. Drought under global warming: a review. WIREs Clim Change. 2:45–65.

[43] Sheffield J , Wood EF, Roderick ML. 2012. Little change in global drought over the past 60 years. Nature. 491:435–438. 23151587 10.1038/nature11575

[44] Körner C , Basler D. 2010. Phenology under global warming. Science. 327:1461–1462. 20299580 10.1126/science.1186473

[45] Sherwood S , Fu Q. 2014. A drier future? Science. 343:737–739. 24531959 10.1126/science.1247620

[46] Huang J , Yu H, Guan X, Wang G, Guo R. 2016. Accelerated dryland expansion under climate change. Nat Clim Change. 6:166–171.

[47] Yuan W , et al 2019. Increased atmospheric vapor pressure deficit reduces global vegetation growth. Sci Adv. 5:eaax1396. 31453338 10.1126/sciadv.aax1396 PMC6693914

[48] Zhou X , et al 2020. Legacy effect of spring phenology on vegetation growth in temperate China. Agric For Meteorol. 281:107845.

[49] Menzel A , Fabian P. 1999. Growing season extended in Europe. Nature. 397:659–659.

[50] Zar JH . 2010. Biostatistical analysis (5th ed.). Englewood Cliffs (NJ): Prentice Hall.

[51] Templ B , et al 2018. Pan European Phenological database (PEP725): a single point of access for European data. Int J Biometeorol. 62:1109–1113. 29455297 10.1007/s00484-018-1512-8

[52] Pinzon J , Tucker C. 2014. A non-stationary 1981–2012 AVHRR NDVI3g time series. Rem Sens. 6:6929–6960.

[53] Buermann W , et al 2018. Widespread seasonal compensation effects of spring warming on northern plant productivity. Nature. 562:110–114. 30283105 10.1038/s41586-018-0555-7

[54] Shen M , et al 2020. Can changes in autumn phenology facilitate earlier green-up date of northern vegetation? Agric For Meteorol. 291:108077.

[55] Chen J , et al 2004. A simple method for reconstructing a high-quality NDVI time-series data set based on the Savitzky–Golay filter. Rem Sens Environ. 91:332–344.

[56] Wang S , Zhang Y, Ju W, Qiu B, Zhang Z. 2021. Tracking the seasonal and inter-annual variations of global gross primary production during last four decades using satellite near-infrared reflectance data. Sci Total Environ. 755:142569. 33038811 10.1016/j.scitotenv.2020.142569

[57] Tagesson T , et al 2021. A physiology-based Earth observation model indicates stagnation in the global gross primary production during recent decades. Glob Change Biol. 27:836–854. 10.1111/gcb.15424 PMC7898396 33124068

[58] Bi W , et al 2022. A global 0.05 degrees dataset for gross primary production of sunlit and shaded vegetation canopies from 1992 to 2020. Sci Data. 9:213. 35577806 10.1038/s41597-022-01309-2 PMC9110750

[59] Beck HE , et al 2022. MSWX: global 3-hourly 0.1 bias-corrected meteorological data including near-real-time updates and forecast ensembles. Bull Am Meteorol Soc. 103:E710–E732.

[60] Cornes RC , van der Schrier G, van den Besselaar EJM, Jones PD. 2018. An ensemble version of the E-OBS temperature and precipitation data sets. J Geophys Res: Atmos. 123:9391–9409.

[61] Liu Q , Fu YH, Liu Y, Janssens IA, Piao S. 2018. Simulating the onset of spring vegetation growth across the northern hemisphere. Glob Chang Biol. 24:1342–1356. 29055157 10.1111/gcb.13954

[62] Kramer K . 1994. Selecting a model to predict the onset of growth of *Fagus sylvatica*. J Appl Ecol. 31:172–181.

[63] Hänninen H . 1990. Modelling bud dormancy release in trees from cool and temperate regions. Acta For Fenn. 0:1–47.

[64] Friedl M , Sulla-Menashe D. 2015. NASA EOSDIS Land Processes DAAC.

[65] Dong L , Wu C, Wang X, Zhao N. 2023. Satellite observed delaying effects of increased winds on spring green-up dates. Rem Sens Environ. 284:113363.

[66] Keenan TF , Richardson AD, Hufkens K. 2020. On quantifying the apparent temperature sensitivity of plant phenology. New Phytol. 225:1033–1040. 31407344 10.1111/nph.16114

[67] Wolkovich EM , et al 2021. A simple explanation for declining temperature sensitivity with warming. Glob Chang Biol. 27:4947–4949. 34355482 10.1111/gcb.15746

[68] Vicente-Serrano SM , Beguería S, López-Moreno JI. 2010. A multiscalar drought index sensitive to global warming: the standardized precipitation evapotranspiration Index. J Clim. 23:1696–1718.

[69] Zhang Y , Keenan TF, Zhou S. 2021. Exacerbated drought impacts on global ecosystems due to structural overshoot. Nat Ecol Evol. 5:1490–1498. 34593995 10.1038/s41559-021-01551-8 PMC8563399

