# Supplementary material for: Non-symmetric responses of leaf onset date to natural warming and cooling in northern ecosystems
Source: PNAS Nexus. 2023 Sep 19;2(9):pgad308. doi: 10.1093/pnasnexus/pgad308 (PMC10538477; doi:10.1093/pnasnexus/pgad308)
Supplement: pgad308_Supplementary_Data [file pgad308_supplementary_data.docx]

Supporting Information for:

Non-symmetric responses of leaf onset date to natural warming and cooling in northern ecosystems

**Supplementary Methods**

**Chilling models**

We used 10 chilling models to measure the number of chilling days^1^. Chilling models C1-C6 were developed based on several specific temperature thresholds. For daily temperatures below 5°C for Model C1 (Eq. 1)^2^, between -10 and 5°C for Model C2 (Eq. 2)^3,4^, between 0 and 5°C for Model C3 (Eq. 3)^2,5^, below 7°C for Model C4 (Eq. 4)^6^, between -10 and 7°C for Model C5 (Eq. 5)^3,6^, and between 0 and 7°C for Model C6 (Eq. 6)^5,6^ the rate of chilling was 1, respectively. The equations for the chilling models C1-C6 are expressed as follows:

$\text{C}_{\text{1}}\text{=}\left\{ \begin{aligned} \text{1 }\text{ T}_{\text{t}}\text{≤}\text{5} \\ \text{0 }\text{ T}_{\text{t}}\text{>5} \end{aligned} \right.$ (1)

$\text{C}_{\text{2}}\text{=}\left\{ \begin{aligned} \text{1}\text{ }\text{-10≤}\text{T}_{\text{t}}\text{ }\text{≤}\text{5} \\ \text{0}\text{ }\text{T}_{\text{t}}\text{>5}\text{ or }\text{T}_{\text{t}}\text{<-10} \end{aligned} \right.$ (2)

$\text{C}_{\text{3}}\text{=}\left\{ \begin{aligned} \text{1}\text{ }\text{0≤}\text{ }\text{T}_{\text{t}}\text{≤}\text{5} \\ \text{0}\text{ }\text{T}_{\text{t}}\text{>5}\text{ or }\text{T}_{\text{t}}\text{<0} \end{aligned} \right.$ (3)

$\text{C}_{\text{4}}\text{=}\left\{ \begin{aligned} \text{1 }\text{ T}_{\text{t}}\text{≤}\text{7} \\ \text{0 }\text{ T}_{\text{t}}\text{>7} \end{aligned} \right.$ (4)

$\text{C}_{\text{5}}\text{=}\left\{ \begin{aligned} \text{1}\text{ }\text{-10≤}\text{T}_{\text{t}}\text{≤}\text{7} \\ \text{0}\text{ }\text{T}_{\text{t}}\text{>7}\text{ or }\text{T}_{\text{t}}\text{<-10} \end{aligned} \right.$ (5)

$\text{C}_{\text{6}}\text{=}\left\{ \begin{aligned} \text{1}\text{ }\text{0≤}\text{ }\text{T}_{\text{t}}\text{≤}\text{7} \\ \text{0}\text{ }\text{T}_{\text{t}}\text{>7}\text{ or }\text{T}_{\text{t}}\text{<0} \end{aligned} \right.$ (6)

where $\text{C}_{\text{i}}$ is the rate of chilling for Models C1-C6, and $\text{T}_{\text{t}}$ is the daily mean temperature (°C).

Model C7, commonly known as the Utah Model, was originally used to calculate the chilling requirements of peach, assigning different weights to various temperature ranges (Eq. (7))^7^. Model C8 was modified from the Utah Model to remove the negative contributions of warm temperatures to accumulated chilling (Eq. (8))^8^.

$\text{C}_{\text{7}}\text{=}\left\{ \begin{aligned} \text{0}\text{ }\text{T}_{\text{t}}\text{≤}\text{1.4} \\ \text{0.5 1.4<}\text{T}_{\text{t}}\text{≤}\text{5} \\ \text{1 2.4<}\text{T}_{\text{t}}\text{≤}\text{9.1} \\ \text{0.5 }\text{9.1<}\text{T}_{\text{t}}\text{≤}\text{12.4} \\ \text{0 }\text{12.4<}\text{T}_{\text{t}}\text{≤15.}\text{9} \\ \text{-0.5 }\text{15.9<}\text{T}_{\text{t}}\text{≤18} \\ \text{-1 }\text{T}_{\text{t}}\text{>18} \end{aligned} \right.$ (7)

$\text{C}_{\text{8}}\text{=}\left\{ \begin{aligned} 0 \text{T}_{\text{t}}\text{≤}\text{1.4} \\ 0.5 \text{1.4<}\text{T}_{\text{t}}\text{≤}\text{2.4} \\ 1 \text{2.4<}\text{T}_{\text{t}}\text{≤}\text{9.1} \\ 0.5 \text{9.1<}\text{T}_{\text{t}}\text{≤}\text{12.4} \\ 0 \text{T}_{\text{t}}\text{>12.4} \end{aligned} \right.$ (8)

where $\text{C}_{\text{i}}$ is the rate of chilling for Models C7-C8, and $\text{T}_{\text{t}}$ is the daily mean temperature (°C).

We employed two triangular models to calculate chilling days, Models C9 and C10 from ref^9^ and ref^10^, respectively.

$\text{C}_{\text{9}}\text{=}\left\{ \begin{aligned} \text{0 }\text{T}_{\text{t}}\text{≤}\text{-3.4 or }\text{T}_{\text{t}}\text{≥10.4} \\ \frac{\text{T}_{\text{t}}\text{+3.4}}{\text{5+3.4}}\text{ -3.4<}\text{T}_{\text{t}}\text{≤}\text{5} \\ \frac{\text{T}_{\text{t}}\text{-10.4}}{\text{5-10.4}}\text{ 5<}\text{T}_{\text{t}}\text{<10.4} \end{aligned} \right.$ (9)

$\text{C}_{\text{10}}\text{=}\left\{ \begin{aligned} \text{0 }\text{T}_{\text{t}}\text{≤}\text{-6.5 or }\text{T}_{\text{t}}\text{≥6.9} \\ \frac{\text{T}_{\text{t}}\text{+6.5}}{\text{6.9-0.2}}\text{ -6.5<}\text{T}_{\text{t}}\text{≤}\text{0.2} \\ \frac{\text{6.9-T}_{\text{t}}}{\text{6.9-0.2}}\text{ 0.2<}\text{T}_{\text{t}}\text{<6.9} \end{aligned} \right.$ (10)

where $\text{C}_{\text{i}}$ is the rate of chilling for Models C9-C10, and $\text{T}_{\text{t}}$ is the daily mean temperature (°C).

**Forcing models**

We used 8 forcing models to measure heat requirement for the spring events of plants^1^. The growing degree days (GDD) models, the most commonly used forcing models, are assumed that there is a linear relationship between the rate of forcing and temperature if the temperature exceeds a specific threshold. Models F1 and F2 adopt temperature thresholds of 0^11,12^ and 5°C^4,13^, respectively. The equations are as follows:

$\text{F}_{\text{1}}\text{=max(}\text{T}_{\text{t}}\text{, 0)}$ (11)

$\text{F}_{\text{2}}\text{=max(}\text{T}_{\text{t}}\text{-5, 0)}$ (12)

where $\text{F}_{\text{i}}$ is the rate of forcing for Models F1-F2, and $\text{T}_{\text{t}}$ is the daily mean temperature (°C).

Some forcing models use maximum instead of mean temperature^1,12^. Models F3 and F4 are based on maximum temperature with thresholds of 0 and 5°C, respectively^1,12^.

$\text{F}_{\text{3}}\text{=max(}\text{Tmax}_{\text{t}}\text{, 0)}$ (13)

$\text{F}_{\text{4}}\text{=max(}\text{Tmax}_{\text{t}}\text{-5, 0)}$ (14)

where $\text{F}_{\text{i}}$ is the rate of forcing for Models F3-F4, and $\text{Tmax}_{\text{t}}$ is the daily maximum temperature (°C).

Model F5 measures the impact of daytime and nighttime temperatures on heat requirement by the weighted average method^14^.

$\text{F}_{\text{5}}\text{=0.25×max(}\text{Tmin}_{\text{t}}\text{-5, 0) + 0.75×}\max\left( \text{Tmax}_{\text{t}}\text{-5, 0} \right)$ (15)

where $\text{F}_{\text{5}}$ is the rate of forcing for Model F5. $\text{Tmin}_{\text{t}}$ and $\text{Tmax}_{\text{t}}$ are the daily minimum and maximum temperatures, respectively.

We used two logistic-function forcing models, i.e., Model F6^9^ and F7^15^.

$\text{F}_{\text{6}}\text{=}\left\{ \begin{aligned} \frac{\text{28.4}}{\text{1+}\text{e}^{\text{-0.185(}\text{T}_{\text{t}}\text{-18.5)}}}\text{ }\text{T}_{\text{t}}\text{>0} \\ \text{0 }\text{T}_{\text{t}}\text{≤0} \end{aligned} \right.$ (16)

$\text{F}_{\text{7}}\text{=}\frac{\text{1}}{\text{1+}\text{e}^{\text{-0.47}\text{T}_{\text{t}}\text{+6.49}}}$ (17)

where $\text{F}_{\text{i}}$ is the rate of forcing for Models F6-F7, and $\text{T}_{\text{t}}$ is the daily mean temperature (°C).

Model F8 is based on a growing degree hour (GDH) model, where plants have an optimum temperature for growth^16^.

$\text{F}_{\text{8}}\text{=}\left\{ \begin{aligned} \text{0 }\text{T}_{\text{t}}\text{≤}\text{T}_{\text{L}}\text{ or }\text{T}_{\text{t}}\text{>}\text{T}_{\text{c}} \\ \frac{\text{T}_{\text{u}}\text{-}\text{T}_{\text{L}}}{\text{2}}\text{(1+cos(π+π}\frac{\text{T}_{\text{t}}\text{-}\text{T}_{\text{L}}}{\text{T}_{\text{u}}\text{-}\text{T}_{\text{L}}}\text{)) }\text{T}_{\text{L}}\text{≥}\text{T}_{\text{t}}\text{≥}\text{T}_{\text{u}} \\ \text{(}\text{T}_{\text{u}}\text{-}\text{T}_{\text{L}}\text{)(1+cos(}\frac{\text{π}}{\text{2}}\text{+}\frac{\text{π}}{\text{2}}\frac{\text{T}_{\text{t}}\text{-}\text{T}_{\text{u}}}{\text{T}_{\text{c}}\text{-}\text{T}_{\text{L}}}\text{)) }\text{T}_{\text{u}}\text{<}\text{T}_{\text{t}}\text{≤}\text{T}_{\text{c}} \end{aligned} \right.$ (18)

where $\text{F}_{\text{8}}$ is the rate of forcing for Model F8, $\text{T}_{\text{t}}$ is the daily mean temperature (°C), $\text{T}_{\text{u}}$ = 25, $\text{T}_{\text{L}}$ = 4, and $\text{T}_{\text{c}}$ = 36.

**References**

1 Wang, H. *et al.* Overestimation of the effect of climatic warming on spring phenology due to misrepresentation of chilling. *Nature Communications* **11**, doi:10.1038/s41467-020-18743-8 (2020).

2 Fu, Y. H. *et al.* Declining global warming effects on the phenology of spring leaf unfolding. *Nature* **526**, 104-107, doi:10.1038/nature15402 (2015).

3 Ritchie, G. A. Effect of freezer storage on bud dormancy release in Douglas-fir seedlings. **14**, 186-190, doi:10.1139/x84-036 (1984).

4 Cannell, M. & Smith, R. J. J. o. a. e. Thermal time, chill days and prediction of budburst in Picea sitchensis. 951-963 (1983).

5 Peaucelle, M. *et al.* Spatial variance of spring phenology in temperate deciduous forests is constrained by background climatic conditions. *Nature Communications* **10**, 5388, doi:10.1038/s41467-019-13365-1 (2019).

6 Weinberger, J. H. in *Proceedings. American Society for Horticultural Science.* 122-128.

7 Arlo Richardson, E., Seeley, S. D. & Walker, D. R. A Model for estimating the completion of rest for Redhaven and Elberta peach trees. *Hortscience* **9**, 331-332, doi:10.21273/HORTSCI.9.4.331 (1974).

8 Luedeling, E., Zhang, M., Luedeling, V. & Girvetz, E. H. Sensitivity of winter chill models for fruit and nut trees to climatic changes expected in California's Central Valley. *Agriculture, Ecosystems & Environment* **133**, 23-31, doi:10.1016/j.agee.2009.04.016 (2009).

9 Hänninen, H. Modelling bud dormancy release in trees from cool and temperate regions. *Acta Forestalia Fennica* **0**, 1-47, doi:doi:10.14214/aff.7660 (1990).

10 Zhang, H., Liu, S., Regnier, P. & Yuan, W. New insights on plant phenological response to temperature revealed from long-term widespread observations in China. *Glob Change Biol* **24**, 2066-2078, doi:10.1111/gcb.14002 (2018).

11 Basler, D. & Körner, C. Photoperiod sensitivity of bud burst in 14 temperate forest tree species. *Agricultural and Forest Meteorology* **165**, 73-81 (2012).

12 Piao, S. *et al.* Leaf onset in the northern hemisphere triggered by daytime temperature. *Nature Communications* **6**, 6911, doi:10.1038/ncomms7911 (2015).

13 Fu, Y. H. *et al.* Increased heat requirement for leaf flushing in temperate woody species over 1980–2012: effects of chilling, precipitation and insolation. *Glob Chang Biol* **21**, 2687-2697, doi:10.1111/gcb.12863 (2015).

14 Fu, Y. H. *et al.* Three times greater weight of daytime than of night-time temperature on leaf unfolding phenology in temperate trees. *New Phytol* **212**, 590-597, doi:10.1111/nph.14073 (2016).

15 Harrington, C. A., Gould, P. J. & Clair, J. B. S. Modeling the effects of winter environment on dormancy release of Douglas-fir. *Forest Ecol Manag* **259**, 798-808 (2010).

16 Anderson, J. L., Richardson, E. A. & Kesner, C. D. in *International Symposium on Computer Modelling in Fruit Research and Orchard Management.*184 edn 71-78 (International Society for Horticultural Science (ISHS), Leuven, Belgium).

**Table S1 LOD response to warming and cooling using satellite-based LOD data from 1998 to 2012**. Grid cells with *p* < 0.05 for temperature changes and partial correlation analysis were retained.

| Biome type | Response to warming (days/°C) | | Response to cooling (days/°C) | | p-value of difference test | Count of warming grid cells | Count of cooling grid cells |
| --- | --- | --- | --- | --- | --- | --- | --- |
|  | Mean | 1SE | Mean | 1SE |  |  |  |
| All | -5.4 | 0.02 | -10.48 | 0.17 | p < 0.001 | 22375 | 1770 |
| Evergreen needleleaf forests | -6.5 | 0.2 | -13.3 | 1.7 | p < 0.001 | 487 | 58 |
| Deciduous needleleaf forests | -4 | 0.06 | -11 | 2.3 | p < 0.05 | 543 | 6 |
| Deciduous broadleaf forests | -8.7 | 0.4 | -10.3 | 0.3 | p < 0.001 | 493 | 236 |
| Mixed forests | -6.1 | 0.1 | -11.9 | 0.6 | p < 0.001 | 1194 | 145 |
| Shrublands | -4.8 | 0.02 | -8.7 | 0.3 | p < 0.001 | 9514 | 304 |
| Woody savannas | -5.1 | 0.07 | -9.6 | 0.5 | p < 0.001 | 2573 | 200 |
| Savannas | -5.6 | 0.05 | -12.4 | 0.8 | p < 0.001 | 3241 | 138 |
| Grasslands | -6.4 | 0.06 | -10.6 | 0.3 | p < 0.001 | 4330 | 683 |

**Table S2 LOD response to warming and cooling using satellite-based LOD data from 1998 to 2012**. Grid cells with *p* < 0.01 for temperature changes and partial correlation analysis were retained.

| Biome type | Response to warming (days/°C) | | Response to cooling (days/°C) | | p-value of difference test | Count of warming grid cells | Count of cooling grid cells |
| --- | --- | --- | --- | --- | --- | --- | --- |
|  | Mean | 1SE | Mean | 1SE |  |  |  |
| All | -5.1 | 0.08 | -11.1 | 0.7 | p < 0.001 | 1938 | 71 |
| Evergreen needleleaf forests | -7.7 | 1.3 | -6.3 | 0.7 | p = 0.37 | 18 | 3 |
| Deciduous needleleaf forests | -4 | 0.1 | -12.9 | 1.4 | p < 0.05 | 35 | 4 |
| Deciduous broadleaf forests | -12.2 | 1.4 | -10.9 | 1 | p =0.47 | 35 | 3 |
| Mixed forests | -11.6 | 1.7 | -10 | 1.8 | p = 0.53 | 24 | 8 |
| Shrublands | -5 | 0.1 | -11.4 | 1.9 | p < 0.01 | 916 | 10 |
| Woody savannas | -5.7 | 0.3 | -10.2 | 0.5 | p < 0.001 | 147 | 17 |
| Savannas | -5.5 | 0.4 | -14.2 | 1.7 | p < 0.05 | 105 | 3 |
| Grasslands | -4.5 | 0.1 | -12 | 1.7 | p < 0.001 | 658 | 23 |

**Table S3 Summary of data used in this study**.

|  | Data | Unit | Spatial resolution | Temporal resolution | Spatial  Range | Source |
| --- | --- | --- | --- | --- | --- | --- |
| LOD | Leaf unfolding | DOY | - | Yearly | Local | PEP725^1^ |
| Climate data | Temperature | °C | 0.1° | monthly | Global | MSWX^2^ |
|  | Precipitation | mm | 0.1° | monthly | Global | MSWX |
|  | Solar radiation | W m^-2^ | 0.1° | monthly | Global | MSWX |
|  | Temperature | °C | 0.1° | daily | Europe | E-OBS^3^ |
|  | Precipitation | mm | 0.1° | daily | Europe | E-OBS |
|  | Solar radiation | W m^-2^ | 0.1° | daily | Europe | E-OBS |
|  | Temperature | K | 0.5° | daily | Global | ISIMIP2b^4^ |
|  | Precipitation | Kg m^-2^ s^-1^ | 0.5° | daily | Global | ISIMIP2b |
|  | Solar radiation | W m^-2^ | 0.5° | daily | Global | ISIMIP2b |
| Spring | NDVI | - | 1/12° | bi-weekly | Global | GIMMS^5^ |
| greening | GPP | g C m^-2^ mon^-1^ | 0.05° | monthly | Global | NIRv GPP^6^ |
| magnitude | GPP | g C m^-2^ d^-1^ | 0.05° | daily | Global | LRF GPP^7^ |
|  | GPP | g C m^-2^ d^-1^ | 0.05° | 8-day | Global | TL-LUE GPP^8^ |
| Land cover | Land cover | - | 0.05° | Yearly | Global | MCD12C1^9^ |

^1^ <http://www.pep725.eu/index.php>

^2^ https://www.gloh2o.org/mswx/

^3^ <https://www.ecad.eu/download/ensembles/download.php>

^4^ <https://data.isimip.org/>

^5^ <https://ecocast.arc.nasa.gov/data/pub/gimms/3g.v1>

^6^ https://doi.org/10.6084/m9.figshare.12981977.v2

^7^ <https://doi.org/10.17894/ucph.b2d7ebfb-c69c-4c97-bee7-562edde5ce66>

^8^ <https://doi.org/10.5061/dryad.dfn2z352k>

^9^ <https://lpdaac.usgs.gov/products/mcd12c1v006/>

**
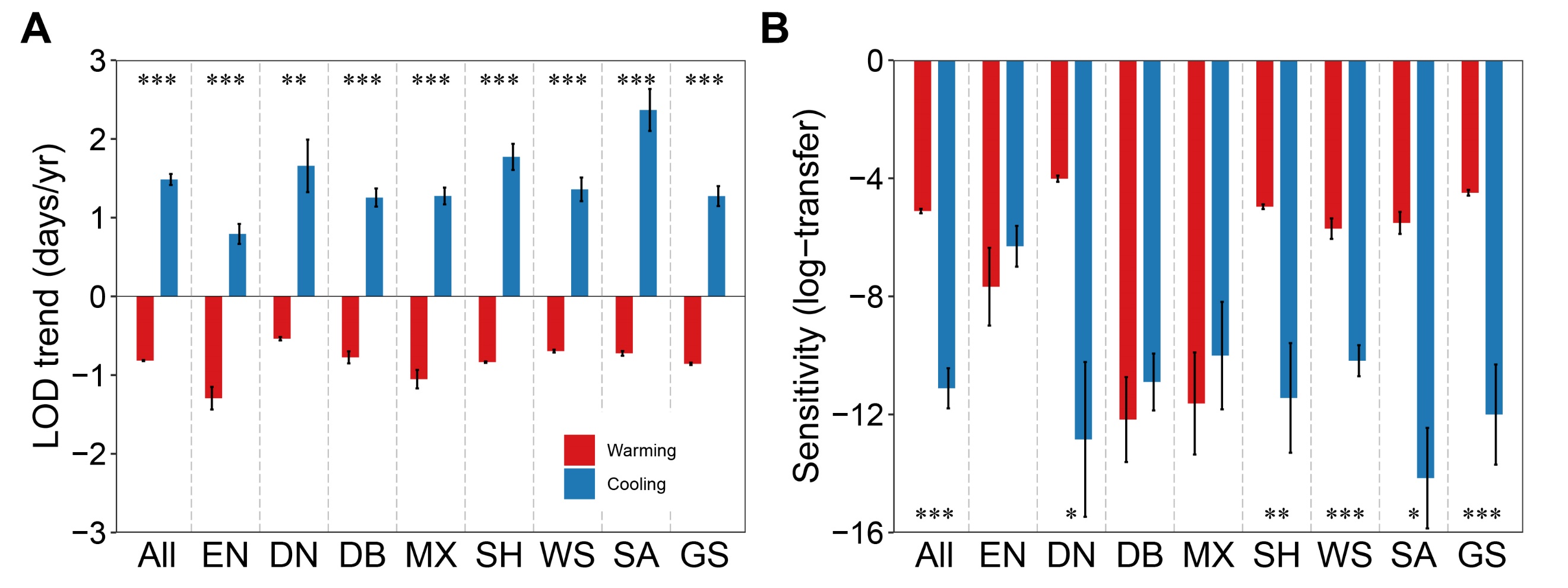
**

**Figure S1** **Comparisons of LOD responses to warming and cooling at the biome scale**. The warming and cooling grid cells were obtained by using *p* < 0.01 for temperature changes and partial correlation analysis. **A**, trends in LOD in warming and cooling areas for biomes from 1998 to 2012. **B**, LOD responses (log-transfer) to warming and cooling at the biome scale from satellite-based LOD data. The bar represents the standard error. Student's t-Test was used to test the significance of difference between the warming and cooling conditions. Significance code for differences: ***, *p* < 0.001; **, *p* < 0.01; and *, *p* < 0.05.


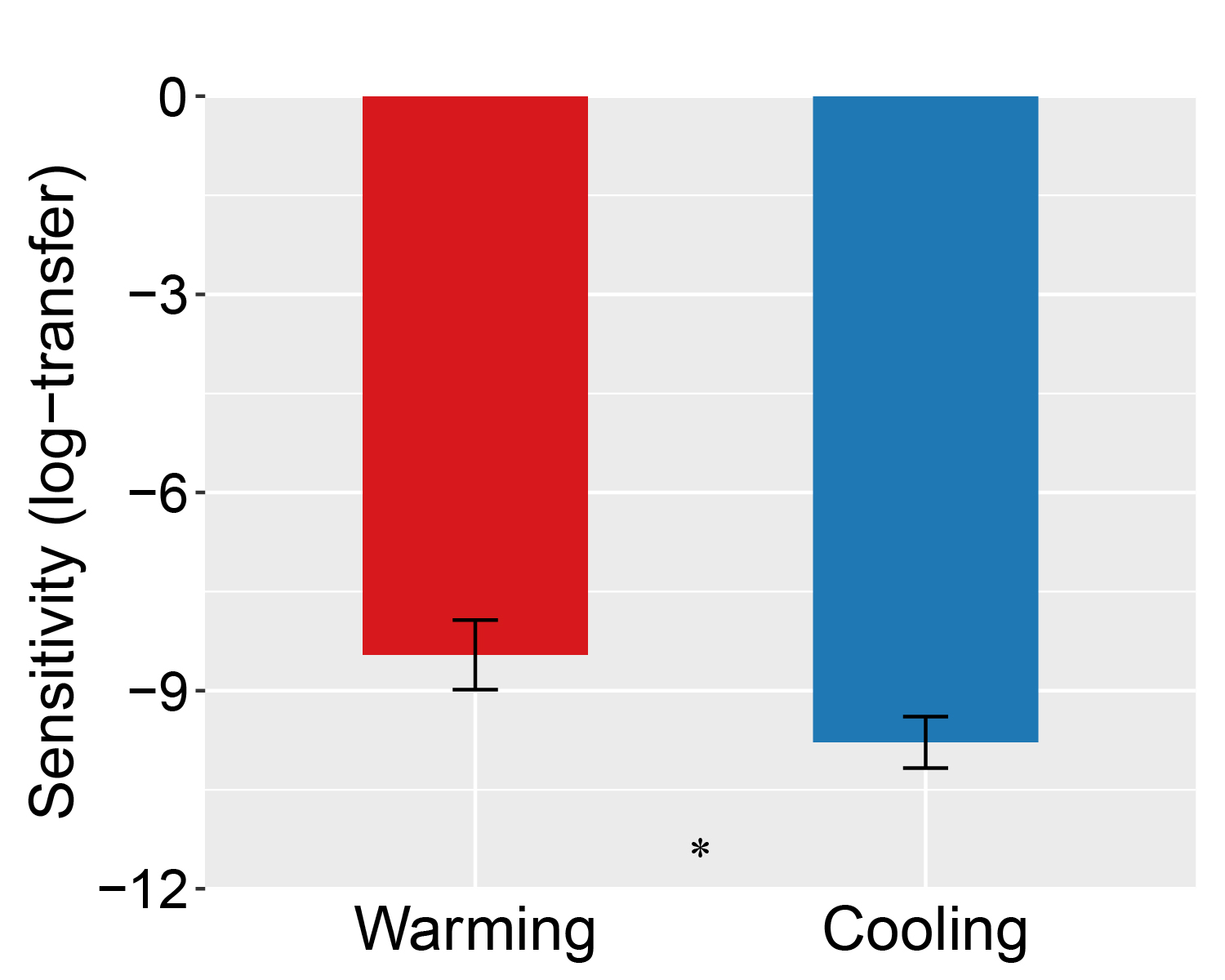


**Figure S2 Comparison between LOD responses to temperature changes in grid cells with warming during 1982-1997 and cooling during 1998-2012**. The bar represents standard error. Significance code for differences: *, p < 0.05.


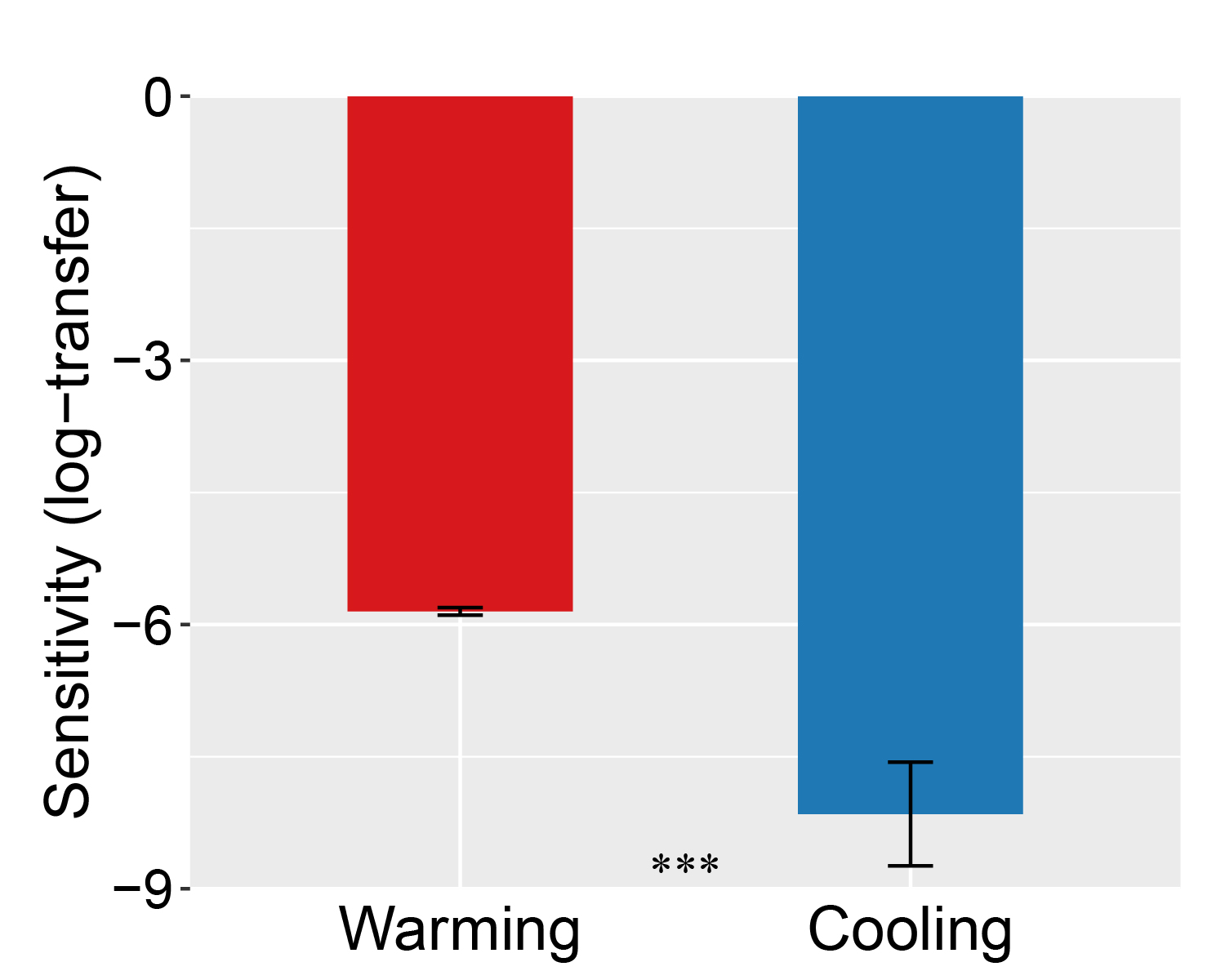


**Figure S3 Comparison between LOD responses to warming and cooling during 1998-2012 using the LOD simulated by a two-phase parallel model (PM)**. The bar represents standard error. Significance code for differences: ***, p < 0.001.

**
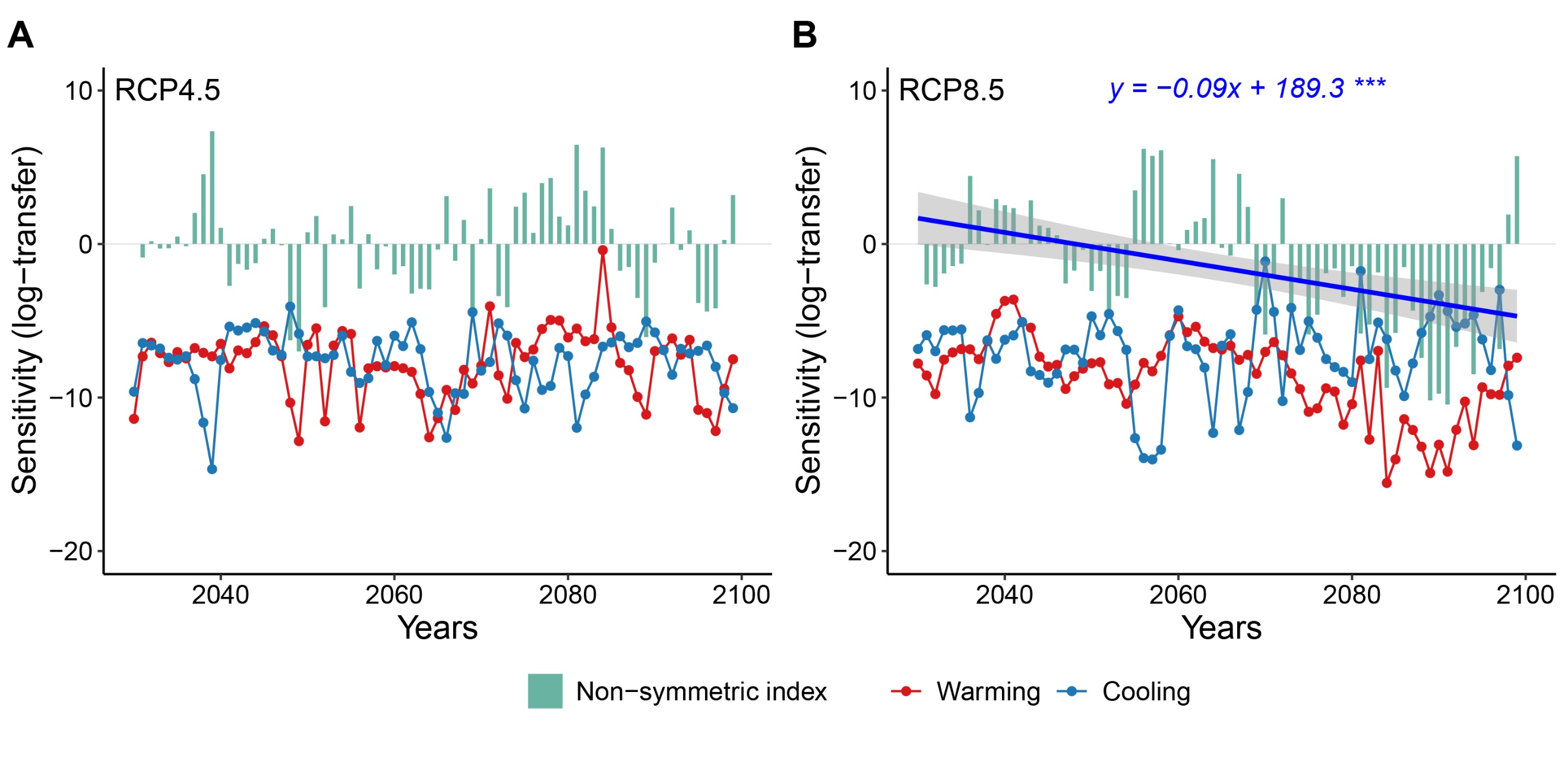
**

**Figure S4 LOD response to natural warming and cooling from 2016 to 2099 under future climate scenarios**. LOD responses with moderate mitigation of carbon emissions (RCP4.5) (**A**) and highest baseline of carbon emissions (RCP8.5) (**B**). The non-symmetric index was defined as the difference between the LOD sensitivities to warming and cooling. The blue line in b indicating the trend of the non-symmetric index was derived from linear regression, and the shown regression equations was significant (*p* < 0.001) estimated using the F-test.

**
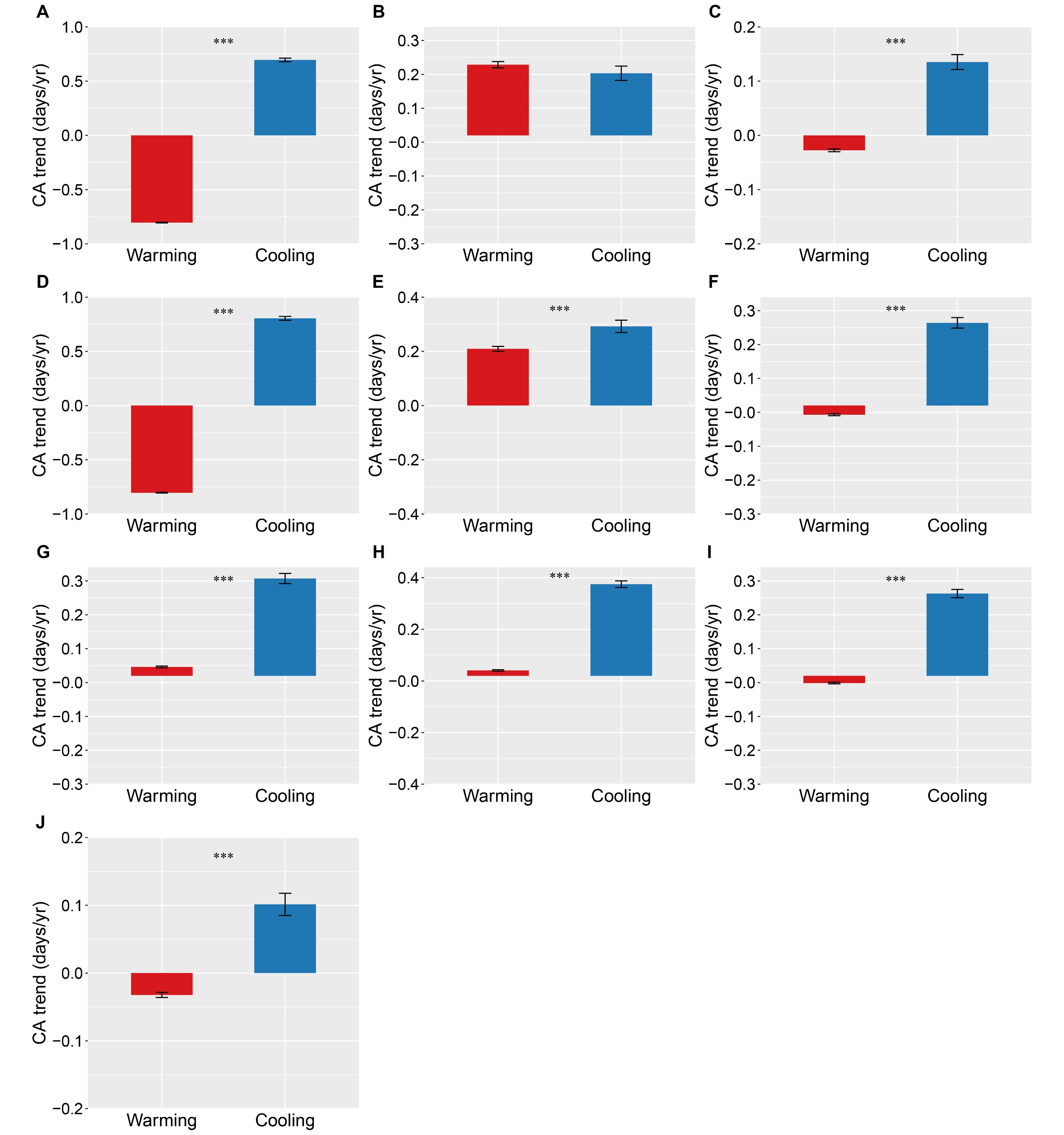
**

**Figure S5 Comparisons of changes for chilling accumulation (CA) in warming and cooling areas obtained from satellite-based analysis during warming hiatus**. **A**-**J** show the results of chilling models C1-10, respectively. The bar represents the standard error. Significance code for differences: ***, *p* < 0.001.

**
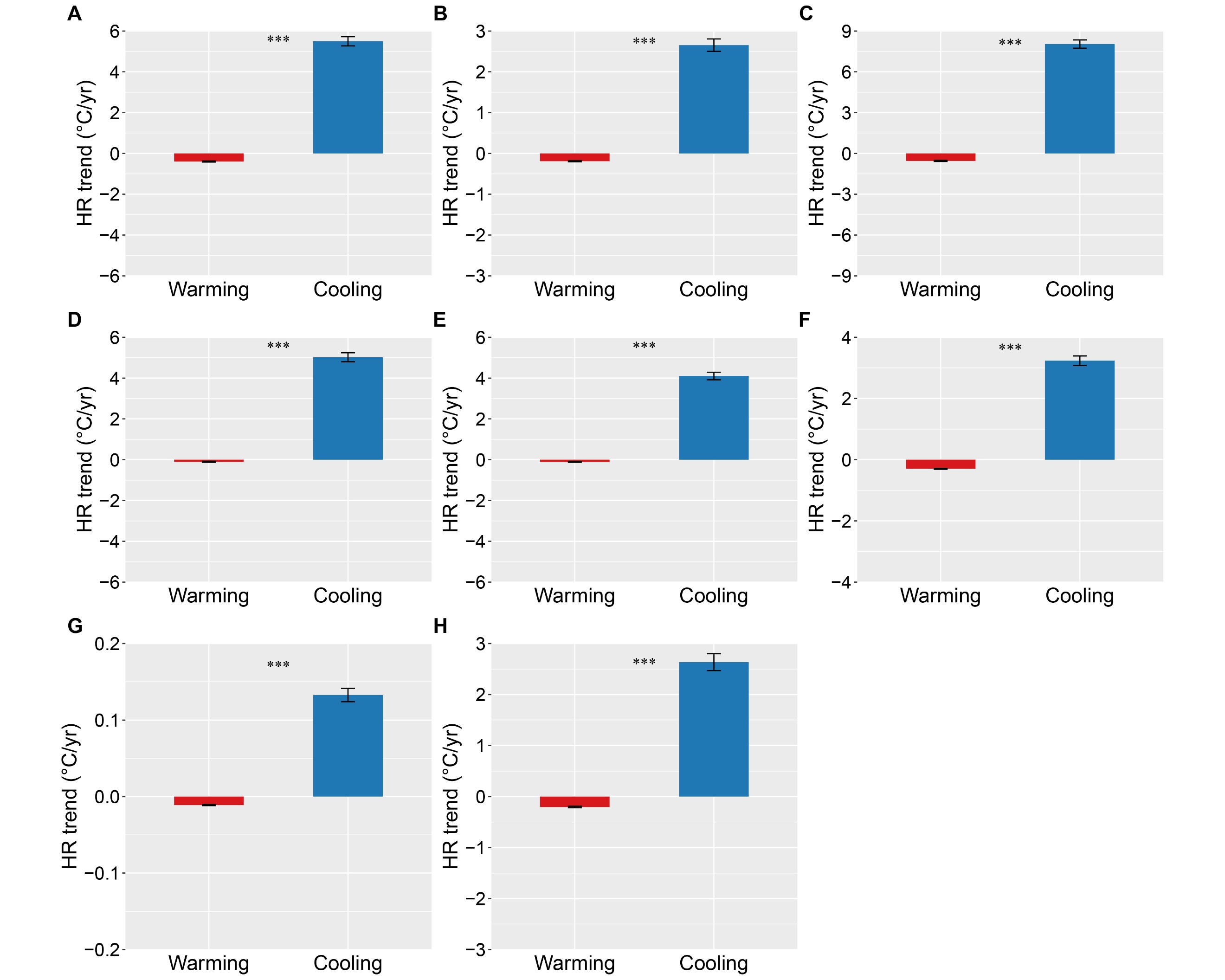
**

**Figure S6 Comparisons of changes for heat requirement (HR) in warming and cooling areas obtained from satellite-based analysis during warming hiatus**. **A**-**H** show the results of forcing models F1-8, respectively. The bar represents the standard error. Significance code for differences: ***, *p* < 0.001.


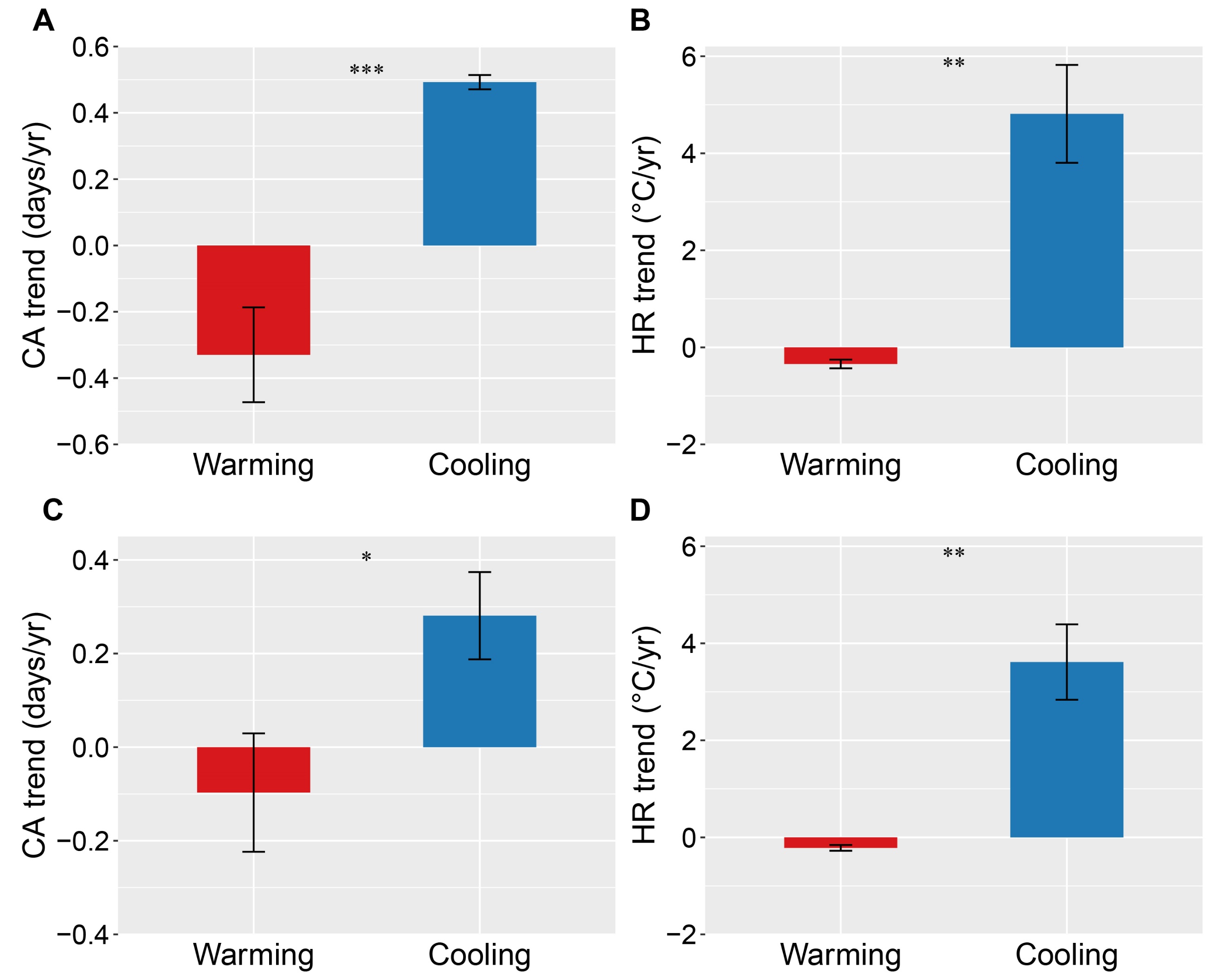


**Figure S7 Comparisons of changes for chilling accumulation (CA) and heat requirement (HR) in warming and cooling areas obtained from satellite-based analysis during warming hiatus**. **A** and **B** present the trends of CA and HR in the warming and cooling areas during 1998-2012 in the biome of trees (forests), respectively. **C** and **D** present the trends of CA and HR in the warming and cooling areas during 1998-2012 in the biome of low vegetation (shrublands, savannas, woody savannas and grasslands), respectively. The bar represents the standard error. Student's t-Test was used to test the significance of difference in the absolute values of trends for CA, HR and SPEI in the warming and cooling areas. Significance code for differences: ***, *p* < 0.001 and **, *p* < 0.01.

**
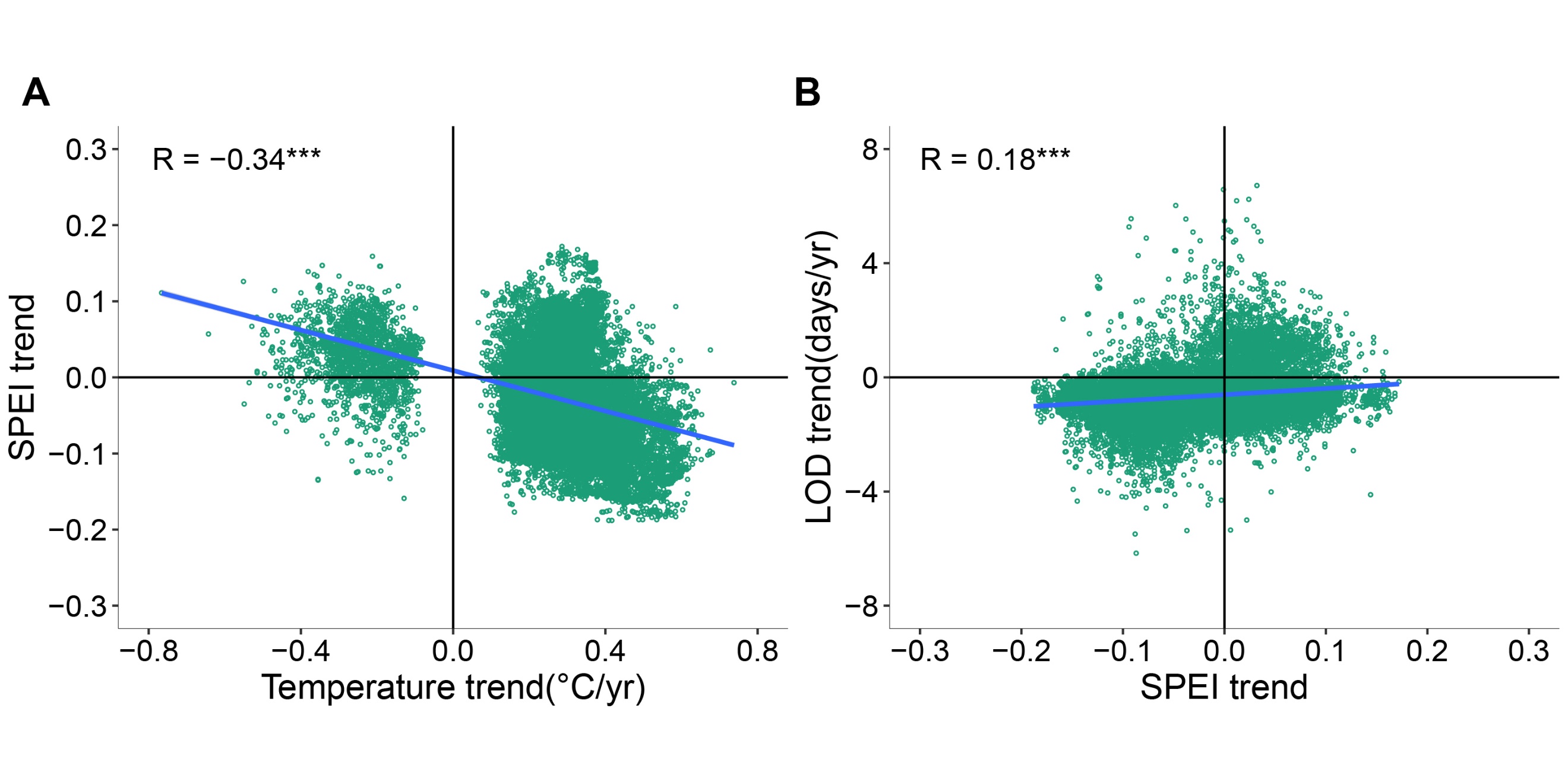
**

**Figure S8 Connections among temperature trend, SPEI trend, and LOD trends**. **A**, relationships between SPEI trend and temperature trend controlling for precipitation and radiation trends. **B**, relationships between LOD trend and SPEI trend controlling for precipitation and radiation trends. Significance code for differences: ***, p < 0.001.

**
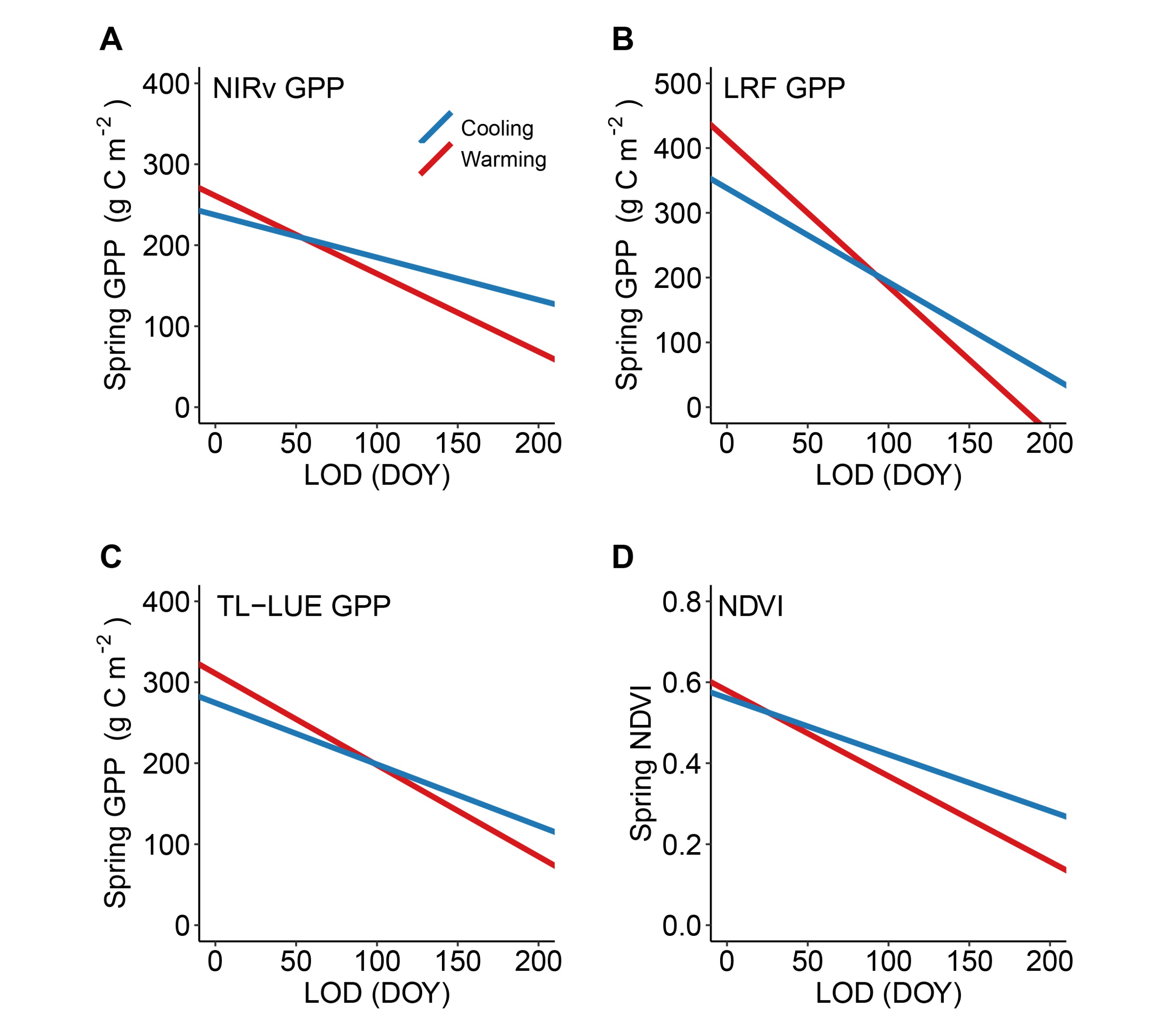
**

**Figure S9 Connections among temperature change, LOD, and spring greening magnitude**. A random slope model was applied to estimate the slopes and intercepts when latitude and longitude were used as random factors performed in the “lme4” package in R4.2.0. **A**-**D**, the comparison for regressions between spring GPP/NDVI and LOD in the warming and cooling areas using NIRV GPP (**A**), LRF GPP (**B**), TL-LUE GPP (**C**), and GIMMS NDVI (**D**), respectively. The spring accumulated GPP and mean NDVI were calculated during the period from March to May.


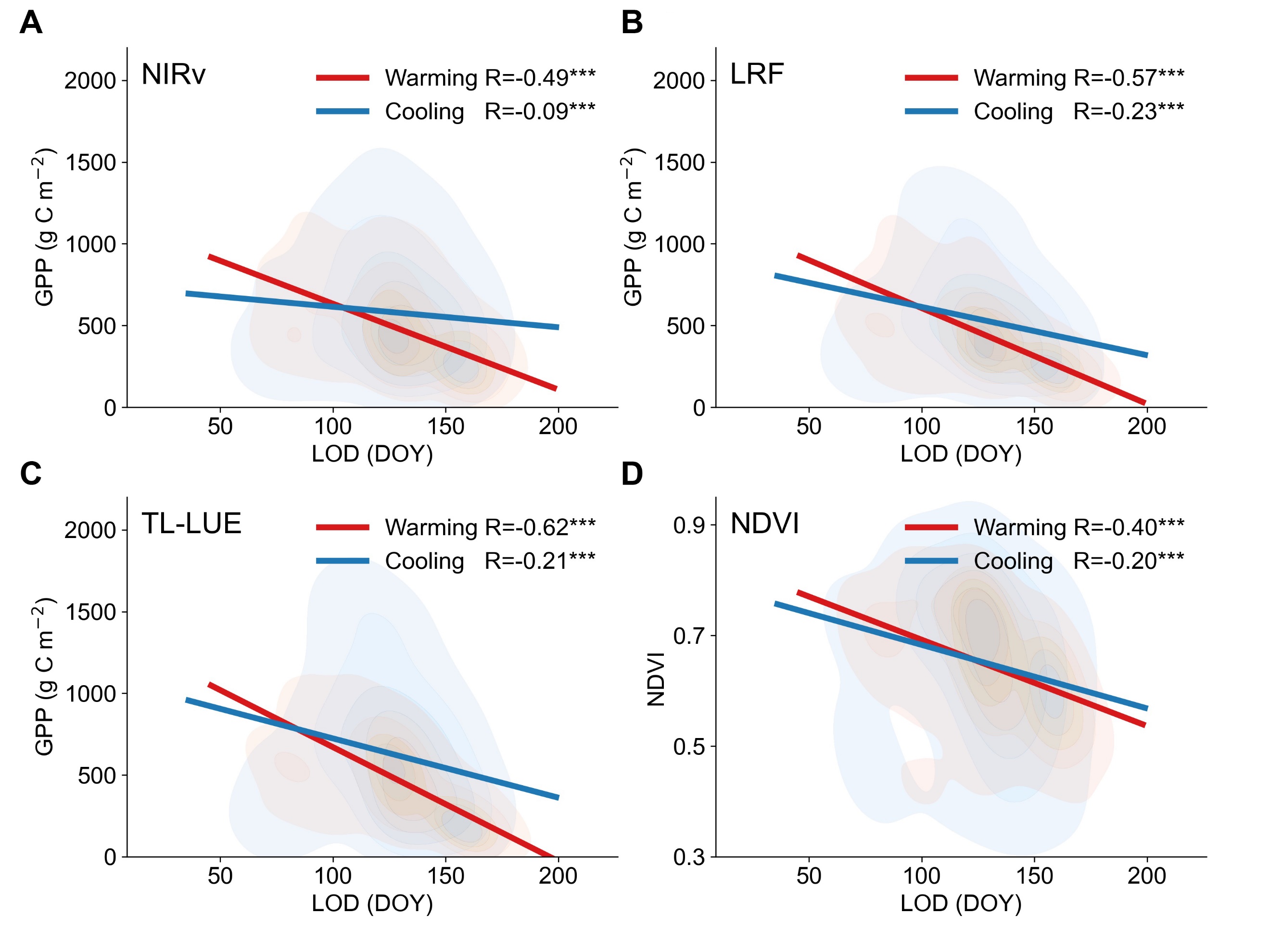


**Figure S10 Connections among temperature change, LOD, and spring greening magnitude**. The comparison for regressions between spring GPP/NDVI and LOD in the warming and cooling areas using the satellite-based near-infrared reflectance (NIRv) GPP (**A**), the light response function (LRF) GPP (**B**), two-leaf light use efficiency model (TL-LUE) GPP (**C**), and Global Inventory Modeling and Mapping Studies (GIMMS) NDVI (**D**), respectively. The spring accumulated GPP and mean NDVI were calculated during the period from the LOD to maturity (i.e., the date corresponding to the maximum NDVI in the GIMMS NDVI3g time series). The red and blue kernel density plots represent the density distribution of warming and cooling grids in GPP/NDVI-LOD space, respectively. The four GPP/NDVI datasets all showed that the differences in the slopes between warming and cooling conditions were significant (*p* < 0.001) by using covariance analysis. Significance code for differences: ***, *p* < 0.001.
